# High expression of Fgr in the left ventricle attenuates myocardial injury in the infarcted region via regulating the phosphorylation level of PI3K/Akt

**DOI:** 10.1042/BSR20253737

**Published:** 2025-10-13

**Authors:** Dongpu Shao, Zhiguo Zhang, Honglei Ji, Lei Shi

**Affiliations:** Department of Cardiology, First Hospital of Jilin University, Changchun, Jilin, 130021, China

**Keywords:** Fgr, PI3K/Akt, myocardial infarction, TWAS, spatial transcriptomics, Single-nucleus RNA sequencing

## Abstract

FGR proto-oncogene (Fgr), a member of the Src family kinases, has garnered attention for its potential involvement in apoptotic signaling, yet its role in cardiovascular diseases, particularly acute myocardial infarction (AMI), remains unexplored. This study sought to investigate whether elevated left ventricular Fgr expression alleviates myocardial injury in the infarcted area and whether this protective mechanism is mediated by modulating phosphoinositide 3-kinase (PI3K)/Akt phosphorylation. The transcriptome-wide association study was initially utilized to screen for susceptibility genes in the left ventricle, with findings validated using bulk-RNA sequencing data from a rat model of left anterior descending coronary artery (LAD) ligation; subsequently, human spatial transcriptomics combined with single-nucleus RNA sequencing data confirmed differential expression of Fgr and PI3K/Akt in the infarcted region. Fgr knockdown via siRNA in H9C2 cells and pharmacological inhibition with TL02-59 in rats were conducted to assess cellular survival and cardiac function, respectively. Fgr emerged as a common candidate gene identified through multi-omics data analysis, with its up-regulation confirmed both *in vivo* and *in vitro*. Fgr silencing in an in vitro oxygenglucose deprivation model significantly reduced cell survival and suppressed PI3K/Akt phosphorylation, whereas TL02-59 administration in rats subjected to LAD ligation impaired post-infarction cardiac function while concurrently inhibiting PI3K/Akt phosphorylation levels. This study demonstrates that Fgr is markedly up-regulated in AMI and exerts cardioprotective effects, possibly through modulation of PI3K/Akt signaling phosphorylation, thereby underscoring its potential as a therapeutic target.

## Introduction

Acute myocardial infarction (AMI) persists as a predominant contributor to global cardiovascular mortality, accounting for >15% of annual cardiovascular-related fatalities worldwide. [1] Epidemiological data reveal that in 2019 alone, approximately 5.8 million new cases of ischemic heart disease were diagnosed across 57 member countries of the European Society of Cardiology. [2] A pronounced disparity in disease burden emerges across economic strata: low- and middle-income countries demonstrate a 77.8% elevation in age-standardized mortality rates compared with high-income regions, a phenomenon driven by population aging trajectories and the expanding pandemic of metabolic syndrome components. [3] Pathophysiologically, AMI represents a time-sensitive vascular emergency characterized by abrupt thrombotic coronary occlusion, precipitating irreversible cardiomyocyte necrosis within 30–60 minutes of sustained ischemia. [4] This initial insult triggers maladaptive systemic inflammation and elevates risks of lethal arrhythmias and hemodynamic collapse. Current data indicate fewer than 30% of eligible patients in remote sites achieve guideline-recommended percutaneous coronary intervention within the 90 minute door-to-balloon window. [5] Clinical trials of coronary reperfusion strategies conducted in developed nations have demonstrated 1 year cardiovascular mortality rates ranging from 2 to 6%. However, real-world large-scale contemporary registries reveal significantly higher rates approaching 11%. [6] These discrepancies emphasize the urgent requirement for additional cardioprotective interventions beyond conventional reperfusion therapies. Although pharmacological adjuncts like antiplatelet agents and beta-blockers provide incremental benefits, these agents fail to mitigate the fundamental pathobiology of progressive ischemic apoptosis. The limitations of this therapeutic modality underscore the imperative to elucidate innovative strategies that specifically target cellular survival pathways. This translational gap not only necessitates a paradigm shift in therapeutic development but also emphasizes the urgency of dissecting molecular mechanisms underlying cell persistence to formulate more efficacious intervention frameworks.

AMI leads to the activation of diverse signaling pathways. Among them, the diminished phosphorylation of phosphoinositide 3-kinase (PI3K)/Akt stands as a characteristic manifestation. [7] An increasing number of studies have revealed that the activation of the PI3K/Akt signaling pathway enables cells to respond to extracellular or intracellular stimuli by regulating survival, proliferation, apoptosis, migration, and other physiological and pathological processes. [8–14] Upon the action of PI3K converting phosphatidylinositol 4,5-bisphosphate into phosphatidylinositol 3,4,5-trisphosphate (PIP3), Akt, the pivotal molecule in the pathway, is activated as PIP3 binds to its pleckstrin homology domain, prompting a conformational change that exposes the Ser473 and Thr308 sites, which are then phosphorylated by phosphoinositide-dependent kinase 1 (PDK1) at Thr308 and PDK2 at Ser473, ultimately regulating cardiac recovery following myocardial infarction (MI) through the downstream signaling cascade. [15–17] The activation or reinstatement of PI3K/Akt phosphorylation holds significant promise in effectively mitigating AMI or the damage caused by myocardial reperfusion [18].

Transcriptome-wide association studies (TWASs) represent an integrative analytical framework that combines individual-level genotypes or genome-wide association study (GWAS) summary statistics with expression quantitative trait loci (eQTL) data to investigate associations between genetically regulated gene expression and complex phenotypes. [19] Unlike conventional GWAS, TWAS adopts a gene-centric paradigm demonstrating tissue-specific resolution while circumventing the multiple testing burden inherent to variant-level analyses. In this investigation, we incorporated GWAS summary statistics for MI and precomputed whole left ventricular myocardial expression prediction models. Utilizing the FUSION software for transcriptome-wide association testing, we systematically prioritized candidate genes implicated in MI pathogenesis. Subsequently, summary-data-based Mendelian randomization (SMR) was applied to integrate eQTL datasets, enabling causal inference between prioritized genes and MI risk. Colocalization analyses were further conducted to evaluate shared causal variants between gene eQTL and MI-associated loci, providing genetic evidence for causal relationships.

To mimic the pathophysiological process of myocardial infarction, we conducted *in vivo* experiments by ligating the left anterior descending coronary artery (LAD) for 24 h and *in vitro* experiments using an oxygen–glucose deprivation model for 12 h. RNA-seq transcriptomic analysis was performed on the apical infarction area of rats to identify differentially expressed genes (DEGs), and these differential genes were intersected with susceptibility genes obtained from TWAS. Ultimately, we preliminarily identified FGR proto-oncogene (Fgr) as a candidate gene. Fgr, a member of the SRC family kinases, has garnered attention due to its potential involvement in cytoskeletal remodeling and apoptotic signaling. Although the functions of Fgr in cancer metastasis and neurodegenerative diseases have been preliminarily explored, its role in cardiovascular diseases, particularly AMI, remains an uncharted territory.

When investigating the pathophysiological changes in MI, cardiac tissue is typically divided into the ischemic zone (IZ), border zone (BZ), and remote zone (RZ). [20] To ascertain the region within the myocardial tissue where the Fgr gene exhibits high expression, we utilized spatial transcriptomic (ST) data and single-nucleus sequencing data from human MI cases. [21] The construction of the ST atlas facilitated the identification of the spatial co-localization of Fgr and PI3K/Akt. Consequently, we formulated the hypothesis that Fgr may enhance cardiomyocyte survival by modulating the activity of the PI3K/Akt signaling pathway. By deconvoluting ST spots into cell-type abundances, we characterized the cellular niches emerging at various stages following MI. Furthermore, leveraging single-cell sequencing data, we determined the expression profiles of Fgr and PI3K/Akt in each cell type. Our multi-omic data-driven approach offers a more comprehensive and multi-faceted understanding of the molecular mechanisms underlying the onset and progression of myocardial infarction.

Fgr knockdown via siRNA in H9C2 cells and pharmacological inhibition with TL02-59 in rats were conducted to assess cellular survival and cardiac function (echocardiography), respectively. This study demonstrates the cardioprotective effect of high-level left ventricular Fgr expression in AMI models through a combination of *in vivo* and *in vitro* experiments. Moreover, it validates that this protective effect is likely mediated by regulating the phosphorylation levels of the PI3K/Akt signaling pathway.

## Materials and methods

### TWAS studies analysis with FUSION

GWAS data of MI were extracted from established cardiovascular genetics repositories, comprising a European-ancestry population (*n*=395,795) with 14,825 confirmed case phenotypes [22]. Genome-wide analysis identifies novel susceptibility loci for MI. The FUSION computational framework (v1.0) was systematically implemented for three interrelated objectives: heritability estimation, polygenic prediction architecture construction, and transcriptome-wide association scanning within the TWAS paradigm. [23] Through systematic implementation of standardized parameters, we processed the aggregated MI genome-wide association dataset using this methodology. To ensure biological relevance, left ventricular myocardial eQTL annotations from the GTEx consortium (version 8) were specifically derived from cardiac left ventricular specimens, forming the foundation for building eQTL-informed predictive frameworks. [24] To evaluate linkage disequilibrium (LD) between the prediction model and single nucleotide polymorphism (SNP) across genomic loci identified by GWAS, we leveraged European population data from the 1000 Genomes Project (https://data.broadinstitute.org/alkesgroup/FUSION/LDREF.tar.bz2). Employing FUSION’s integrated post-processing module, we conducted locus-specific analyses to distinguish whether multiple significant genes within a genomic region represented independent association signals or functional interactions. This involved constructing joint models incorporating all significant genes at each TWAS-defined locus to identify robust association patterns.

The framework was systematically applied to each myopia-associated GWAS locus by iteratively testing one SNP at a time while maintaining other variables in the model. Genes demonstrating persistent significance across these joint analyses were classified as jointly significant, whereas those losing significance were designated marginally associated. To validate TWAS findings, we implemented a dual-criterion approach combining FUSION with COLOC colocalization analysis, requiring both *P*<0.05 and posterior probability PP4>0.95 to establish credible evidence of shared causal variants between gene expression and myopia traits. This stringent threshold ensured that only genes meeting both statistical significance and colocalization standards were prioritized as candidate susceptibility loci for myopia pathogenesis.

### SMR

To prioritize susceptibility genes from TWAS-positive candidates, we applied the SMR framework. SMR analysis was performed to identify genes demonstrating causal associations with MI, enabling prioritization of functionally relevant loci. Input data were preprocessed following official guidelines to ensure compatibility with SMR requirements. Analyses were executed using default parameters specified in the SMR software documentation (https://yanglab.westlake.edu.cn/software/smr/#SMR), adhering to protocols established by the Yang Lab. [25] eQTL meeting the significance threshold of *P*<0.05 were considered causally linked to myopia pathogenesis. Additionally, HEIDI test results with *P*>0.05 were interpreted as absence of pleiotropic effects, reinforcing the specificity of identified gene-trait associations.

### Bayesian colocalization

To evaluate colocalization between eQTL and MI, we implemented Bayesian statistical frameworks using the ‘coloc’ package in R (v5.1.0, accessible via cran.r-project.org/web/packages/coloc) [26]. A stringent prior probability threshold of 1 × 10⁻⁶ was established to assess the likelihood that a random genetic variant would exhibit causal associations with both eQTL and MI GWAS datasets. Colocalization was stringently defined as the presence of a shared causal variant supported by posterior probability criteria: either PP4≥0.75 or the sum of PPH3 and PPH4 exceeding 0.8. [27] These dual-threshold criteria ensured robust identification of genetic loci where expression-trait associations and myopia susceptibility shared common biological determinants, enhancing the reliability of causal inference from colocalization analyses.

### Bulk RNA-seq data

RNA was extracted from the left ventricular apex tissue of three rats randomly selected from the sham or MI groups. Approximately 1 ug of total RNA was utilized as input for complementary DNA (cDNA) library preparation, which was accomplished using an Illumina TruSeq kit, followed by sequencing on an Illumina NovaSeq platform. The raw sequencing reads underwent further processing using the specialized bioinformatics platform BMKCloud (www.biocloud.net). Genes were identified as differentially expressed if they had a false delivery rate (FDR) adjusted *P*-value below 0.01 and an absolute log2 fold change exceeding 2.

### Download and analysis of human cardiac snRNA-seq data

The snRNA-seq and ST data processed by the authors were downloaded from the cellxgene website (https://cellxgene.cziscience.com/collections/8191c283-0816-424b-9b61-c3e1d6258a77). [21] Samples were selected based on the criteria of having a disease status of AMI with days after infarction ≤5 for three samples from IZ regions (IZ_P9, IZ_P9_rep2, IZ_P3) and two samples from BZ regions (BZ_P2, BZ_P3). Additionally, samples with a disease status of AMI and days after infarction >10 were selected, including two samples from IZ regions (IZ_P15, IZ_P15_rep2), along with four normal control samples (control_P1, control_P7, control_P8, control_P17). The R package Seurat (version 5.0.3) was utilized for the analysis of snRNA data. Low-quality cells were filtered out based on the criteria of nFeature_RNA>300 & nFeature_RNA<7000 & percent.mt<5, resulting in a final dataset of 83,582 cells. Subsequently, the NormalizeData function was applied for data normalization, FindVariableFeatures was used to identify highly variable genes (nFeature=2000), and ScaleData were employed for data scaling. Linear dimensionality reduction was performed using RunPCA, and batch effects were removed using RunHarmony. The RunUMAP function was then used to perform uniform manifold approximation and projection (UMAP) dimensionality reduction on the top 30 dimensions. Finally, cells were clustered and subpopulations were identified using the FindNeighbors function (dims=1:30) and the FindClusters function (resolution=0.3). Eleven primary cell types, including lymphocytes, myeloid cells, mast cells, proliferating cells, endothelial cells, pericytes, vascular smooth muscle cells, fibroblasts, adipocytes, neurons, and cardiomyocytes, were defined using classical marker genes. The single-cell dataset with defined cell types was used as a reference dataset to assist in identifying the cellular distribution within the ST data. The FindMarkers function was utilized to analyze DEGs between IZ vs. control (CTRL) and BZ vs. CTRL comparisons. The threshold for identifying DEGs was set as follows: *P*-value<0.05 and |log₂FC|>0.

### Spatial transcriptomics data analysis

ST data were also processed using the R package Seurat. For each sample individually, we performed the following analyses: data normalization, scaling, and identification of highly variable features (nFeature=3000) were conducted using the SCTransform function. Pathway scores were computed using the AddModuleScore function. Correlation coefficients and *P*-values for key genes were calculated within each sample. Subsequently, the R package spacexr (version 2.2.1) was employed to annotate cell types within the ST data. This package utilizes the ‌robust cell-type decomposition (RCTD)‌ algorithm, a deconvolution-based approach that leverages snRNA-seq data as a reference to resolve the cellular composition (proportions of cell types) within each spatial spot. Following the creation of an RCTD object linking the single-nucleus RNA-seq reference to the spatial data using the CreateRCTD function, the RCTD algorithm was executed via the RunRCTD function with the parameter doublet_mode=‘full.’ For visualizing the cell-type proportions identified by the RCTD algorithm, the R package STdeconvolve (version 1.9.0) was utilized to generate scatter pie charts.

### Integrated analysis of spatial transcriptomics data

‌Integrated ST analysis was performed using the Seurat R package. Expression matrices from 11 samples were merged via RowMergeSparseMatrices, yielding a combined dataset of 42,834 spots. Data processing included normalization, scaling, and identification of highly variable features (nFeature=3000) using SCTransform; linear dimensionality reduction via RunPCA; batch correction across samples using RunHarmony; non-linear dimensionality reduction through RunUMAP on the first 30 principal components; and clustering via FindNeighbors and FindClusters (resolution=0.3) resulting in 13 clusters (0–12). Clusters were subsequently categorized into five groups (C1–C6) based on cell-type-specific marker genes from snRNA data: C1 primarily contained cardiomyocytes exclusively expressing cardiomyocyte markers; C2 comprised cardiomyocytes, myeloid cells, fibroblasts, and endothelial cells with concurrent high expression of all four lineages’ markers; C3 contained cardiomyocytes, myeloid cells, and endothelial cells showing no significant differential expression of respective markers; C4 predominantly contained myeloid cells and cardiomyocytes with lymphocyte enrichment and corresponding marker expression; C5 mainly contained myeloid cells and fibroblasts co-expressing both lineages’ markers; and C6 exclusively contained vascular smooth muscle cells (VSMCs) specifically expressing VSMC markers. Cluster-specific DEGs were identified using FindAllMarkers with thresholds (avg_log2FC>0.5 & p_val_adj<150 & pct.1>0.3 & pct.2<0.3 & diff.pct>0.2), with the top five DEGs ranked by highest diff.pct values visualized in bubble plots. For the purpose of identifying DEGs, the threshold criteria were established as a *P*-value of less than 0.05 and |log₂FC| greater than 0.

### Analysis of cardiomyocyte subpopulations

After extraction, cardiomyocytes were re-processed using the ‘FindVariableFeatures’ function to identify highly variable genes. Principal component analysis (PCA) was then performed using the ‘RunPCA’ function, followed by batch effect removal with the ‘RunHarmony’ function. UMAP dimensionality reduction was applied to the top 20 dimensions using the ‘RunUMAP’ function. Subsequently, cell clustering and subpopulation identification were conducted using the ‘FindNeighbors’ function (with ‘dims=1:20’) and the ‘FindClusters’ function (with a resolution of 0.25). To further refine the clustering, the ‘FindNeighbors’ and ‘FindClusters’ functions were utilized again (with a resolution of 0.3), resulting in a total of five clusters, labeled as CM1 to CM5. The `FindAllMarkers` function (with parameters ‘only.pos=T’, ‘min.pct=0.2’, and ‘min.diff.pct=0.2’) was employed to identify DEGs among the cardiomyocyte subpopulations. The top five genes with the highest diff.pct values (pct.1-pct.2) were selected as marker genes for each subpopulation, and a bubble plot was generated to visualize these findings.

Bar plots and violin plots were generated using the R package ‘ggplot2’ (version 3.5.0). Statistical tests were conducted using the R package ‘ggpubr’ (version 0.6.0), with the ‘geom_signif’ function employed to calculate the *P*-values for t-tests comparing IZ vs. CTRL and BZ vs. CTRL. Significance levels were indicated as follows: * for *P*-value<0.05, ** for *P*-value<0.01, **** for *P*-value<0.001, and ns for *P*-value>0.05.

### Reverse transcription quantitative real-time PCR

Total RNA was isolated from rat heart tissue using TRIzol Reagent (Thermo Fisher Scientific, #15596026) following the manufacturer’s instructions. The extraction process involved homogenizing the tissue in TRIzol, adding chloroform, and then centrifuging after gentle mixing to separate the aqueous phase. RNA was precipitated with isopropanol, pelleted by centrifugation, washed with 75% ethanol, and collected by another centrifugation step. The RNA pellet was resuspended in diethyl pyrocarbonate-treated water. RNA purity was assessed by measuring the optical density ratio at 260 nm/280 nm. Subsequently, RNA was reverse transcribed into cDNA using oligo(dT) primers. Quantitative PCR (qPCR) was performed using gene-specific primers and SYBR Premix Ex Taq. All qPCR reactions were run in triplicate, with GAPDH serving as the internal control. The primer sequences used for real-time RT-qPCR were as follows: Fgr-F (Rat): 5′- CAGAGGCGACCACATAAAGCA -3′, Fgr-R (Rat): 5′- TTGCTGTAGCCAAATTCGTTG -3′; Gapdh-F (Rat): 5′- ACAGCAACAGGGTGGTGGAC -3′, Gapdh-R (Rat): 5′- TTTGAGGGTGCAGCGAACTT -3′.

### Western blot analysis

Protein levels in H9c2 cells and rat heart tissues were quantified using the BCA Protein Assay Kit (Beyotime Institute of Biotechnology, P0010). Proteins were then resolved by 12% SDS-PAGE and transferred to a PVDF membrane. The membrane was blocked with 5% nonfat milk and incubated overnight at 4°C with primary antibodies against FGR (1:1000; HUABIO, #ER62997), PI3K (1:1000; Selleck, #F1720), p-PI3K (1:1000; Zenbio, #690398), Akt (1:1000; Selleck, #F0004), p-Akt (1:1000; Selleck, #F0699) and GAPDH (1:5000; Proteintech, #60004–1-Ig). Following incubation with the primary antibodies, the membrane was exposed to the corresponding secondary antibody (1:5000; Proteintech) for 1 h. Protein bands were visualized using ECL chemiluminescence (Epizyme Biotech, #SQ202) and analyzed with ImageJ software.

### Assessment of cell viability

Cell proliferation was assessed using the Cell Counting Kit-8 (CCK-8) assay (DOJINDO, #CK04). H9c2 cells were plated in 96-well plates (5 × 10³ cells/well) for 24 h adhesion prior to oxygen–glucose deprivation (OGD) induction‌. Following treatment, 10 μL CCK-8 reagent was administered to each well and incubated for 2 h under light-protected conditions at 37°C. Absorbance at 450 nm was quantified using a microplate reader to determine cellular viability‌.

### Cell culture and the OGD model

The H9c2 cardiomyoblast cell line (rat embryonic origin) was procured from HyCyte. Cells were maintained in high-glucose DMEM supplemented with 10% fetal bovine serum (FBS) and 1% penicillin/streptomycin under standard culture conditions (37°C, 5% CO2, humidified atmosphere). For OGD model establishment, H9c2 cells were seeded in 96-well plates at 5 × 10³ cells/well and allowed to adhere for 24 h. The culture medium was then replaced with glucose-free and serum-free DMEM (Wuhan Pricella Biotechnology, #PM150280), followed by exposure to hypoxic conditions (1% O2, 5% CO2, and 94% N2) for 12 h using a tri-gas incubator to simulate ischemic stress.

### Animal experiments

All animal procedures were performed in compliance with the National Institutes of Health Guidelines for Laboratory Animal Welfare and received dual oversight from both the Institutional Animal Care and Use Committee and the Animal Experimental Ethics Committee of Jilin University First Hospital (Ethical Approval No. 20250307–18). Experimental subjects were maintained in a specific pathogen-free environment with controlled temperature (22 ± 1°C), relative humidity (55 ± 5%), and a standardized 12-h photoperiod. Throughout the study duration, animals received standardized rodent chow and sterilized water *ad libitum*, with environmental enrichment provisions to ensure species-typical behavior expression. Male Wistar rats, with body weights controlled within the range of 230–250 g per rat, were subjected to a one-week adaptive feeding period beforehand. Both the feeding of rats and the subsequent establishment of the MI model were carried out at the Animal Experiment Center of the School of Basic Medical Sciences, Jilin University.

### Establishment of the MI injury model

The rats were anesthetized via inhalation of 1–2% isoflurane, followed by tracheal intubation with a ventilator (BME-ZH, ZH-DW-3000A/B). After removing the hair from the chest and neck regions, the surgical site was disinfected with iodophor. A surgical incision was made along the left subclavian midline at the fourth intercostal space to access the heart. A 6/0 nylon suture was placed around the LAD artery to create a slipknot, inducing 24-h myocardial ischemia. Rats in the sham group underwent the same surgical procedures without artery ligation. The chest cavity was closed immediately after the surgery. The tracheal tube was carefully removed, and the rats were placed under a nose cone providing 100% oxygen. Throughout the surgical procedure and recovery period, the animals were maintained on a 38°C heating mat to ensure stable body temperature.

### Euthanasia and tissue collection

After the blood samples had been collected, the rats were humanely euthanized by administering an overdose of isoflurane (3–4%). Following this, myocardial tissues were harvested from the infarcted regions. In the case of the sham-operation group, myocardial tissues were obtained from corresponding anatomical sites, mirroring those collected from the MI model group, to serve as controls.

### Inhibitor administration

TL02-59, an Fgr inhibitor, was obtained from MedChemExpress (Monmouth Junction, NJ, U.S.A.). The compound was prepared in a solution containing 45% saline, 10% DMSO, and 40% PEG300. Both TL02-59 at a dose of 10 mg/kg and the vehicle were administered intravenously via the tail vein once, 1 h prior to MI [28,29].

### Histopathological examination

At 24 h following ligation of the LAD, the left ventricles of the hearts were collected and immediately rinsed with phosphate-buffered saline (PBS). Subsequently, they were fixed in 4% polyformaldehyde for 24 h. The specimens were then embedded in paraffin and carefully sectioned into 4-micrometer-thick slices, which were stained with hematoxylin and eosin. These stained sections were thoroughly examined under a Nikon light microscope in Japan.

### Echocardiography measurement

By 24 h after ligation of the LAD artery, cardiac function was evaluated in rats under sedation (2% isoflurane). Critical parameters of left ventricular performance, including left ventricular internal diameter at diastole (LVIDd), left ventricular internal diameter at systole (LVIDs), left ventricular fractional shortening (LVFS), and left ventricular ejection fraction (LVEF), were automatically acquired and computed using a 10 S scan head.

### Fgr small interfering RNA transfection

To examine the functional consequences of Fgr silencing, H9C2 cardiomyocytes were transfected with either control siRNA (forward: 5′-UUCUCCGAACGUGUCACGU-3′; reverse: 5′-ACGUGACACGUUCGGAGAA-3′) or Fgr-specific siRNA (forward: 5′-GGAAGUGAAUGACGGUCUA-3′; reverse: 5′-UAGACCGUCAUUCACUUCC-3′) using RNATransMate transfection reagent (E607402-0500, Sangon Biotech, China). Cells were systematically categorized into four experimental cohorts: (1) Control+si-NC; (2) Control + si-Fgr; (3) OGD 12 h + si-NC; and (4) OGD 12 h + si-Fgr.

### Statistical analysis

The figures indicate the number of samples (n) employed in each experiment, denoting biological replicates. Results are presented as the mean ± standard deviation. Statistical analyses were executed using GraphPad Prism version 9.5.0 (GraphPad Software, United States). For comparisons between two groups, Student’s t-test was employed, while for comparisons among multiple groups, an ordinary one-way ANOVA was performed, succeeded by Dunnett’s multiple comparisons test. Data are expressed as mean ± s.d., with statistical significance defined as *P*<0.05. Asterisks in the figures denote levels of statistical significance (*P<0.05, **P<0.01, ***P<0.001, ****P<0.0001), as elaborated in the figure legends.

## Results

### Identification of genes in left ventricular tissue with causal relationships to MI in humans via TWAS analysis

**Figure 1:**
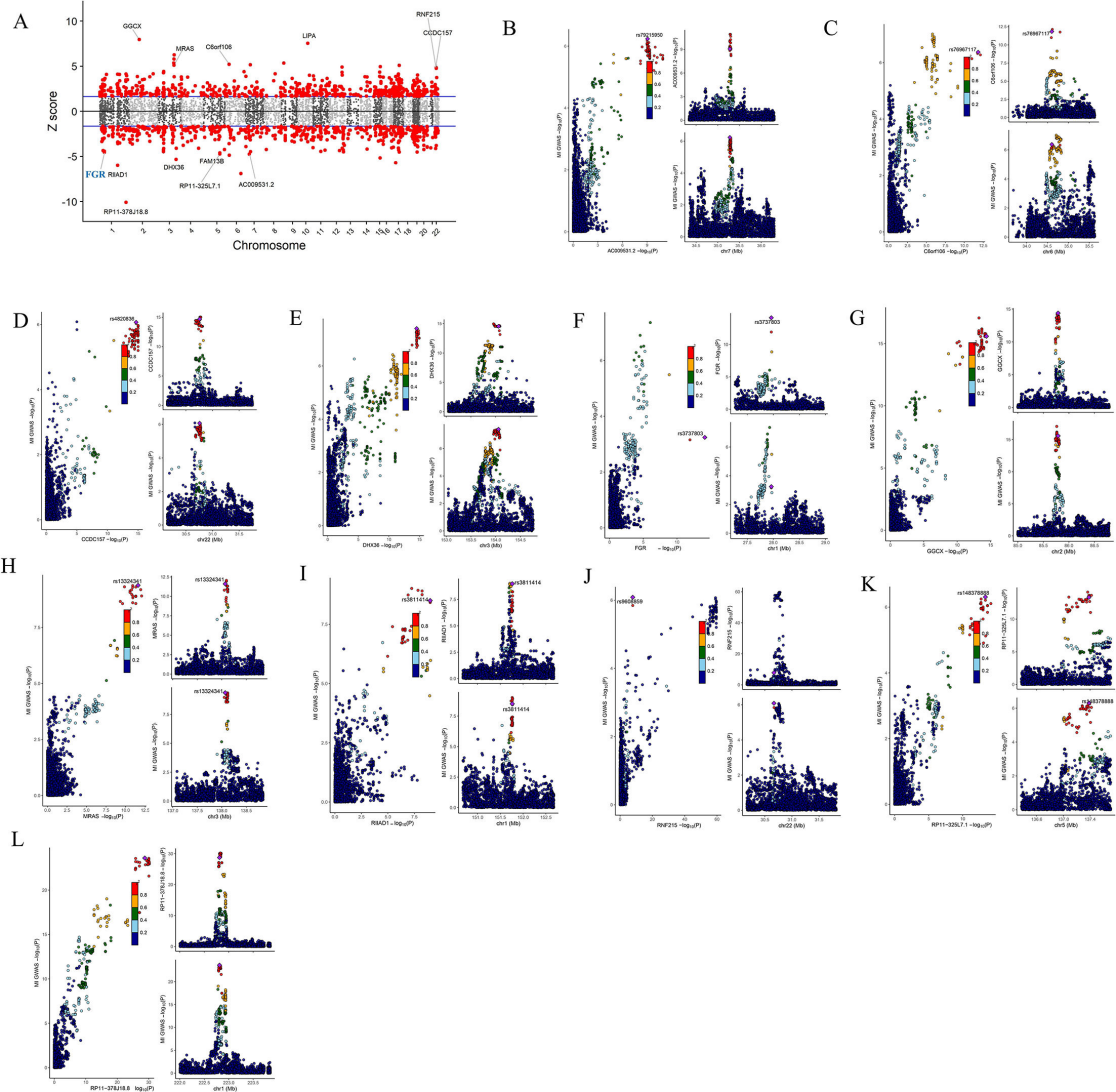
TWAS identifies left ventricle-specific susceptibility genes for myocardial infarction using eQTL data. A, the results of the TWAS analysis. B–L, colocalization results between eleven genes and myocardial infarction.

The predictive models for left ventricular tissue encompassed 5994 genes (30.24%) exhibiting significant *cis*-heritability estimates (*P*<0.01) and corresponding trained weights. Using the FUSION pipeline, gene expression components associated with MI were identified. This led to the discovery of 811 susceptibility genes in the left ventricular tissue (Figure 1A). Utilizing the results of approximate colocalization analysis obtained via the TWAS algorithm and based on a PPH4 threshold of ≥0.95, a total of 13 genes were ultimately identified as myocardial infarction susceptibility genes (Figure 1A). Among these, six genes (MRAS, LIPA, C6orf106, CCDC157, GGCX, RNF215) demonstrated a positive correlation with MI, while the remaining seven genes (RP11-378J18.8, Fgr, RIIAD1, DHX36, RP11-325L7.1, AC009531.2, FAM13B) exhibited protective effects against the disease (Figure 1A). To establish a causal relationship between these 13 genes and myocardial infarction, we employed the SMR method. After Bonferroni correction, the results revealed that, with the exception of the FAM13B gene (no corresponding data available) and the LIPA gene (unstable causal relationship with MI), the remaining 11 genes all demonstrated a causal relationship with myocardial infarction (Table 1). Furthermore, the effect sizes were consistent with those obtained from the TWAS analysis. During the HEIDI test, two genes indicated heterogeneity in the SMR results. It is important to note that a spurious SMR result may emerge from a locus where the associations between SNP-exposure and SNP-outcome are attributed to two distinct causal SNPs in close LD. When an SNP is significantly associated with both exposure and outcome, colocalization analysis can be utilized to further investigate this phenomenon. Evidence suggests that proteins supported by both SMR and colocalization findings are more likely to serve as successful drug targets, potentially reinforcing the notion that GWAS-nominated drug targets have a higher probability of approval. Leveraging eQTLs from left ventricular tissue data, we possessed sufficient statistical power (PPH3 + PPH4≥0.80) to conduct a colocalization analysis for 11 genes (Figure 1B–L, Table 2). Among these, MRAS, C6orf106, CCDC157, GGCX, RNF215, RP11-378J18.8, RIIAD1, DHX36, RP11-325L7.1, and AC009531.2 exhibited strong evidence of colocalization (PPH4≥0.75) (Figure 1B–L, Table 2). In conclusion, by integrating TWAS analysis, SMR analysis, and colocalization analysis, we successfully identified 11 genes that exhibit a causal relationship with myocardial infarction.

**Table 1:** The results of analyzing the causal relationship between genes and MI using the SMR method

Exposure (Gene)	Outcome (Disease)	Beta (SMR)	SE (SMR)	*P*-value (SMR)	*P*-value (HEIDI)	NSNP (HEIDI)
MRAS	MI	0.260908	0.056333	3.63E-06	9.05E-01	8
RP11-378J18.8	MI	-0.11726	0.015472	3.49E-14	1.60E-01	20
LIPA	MI	0.07683	0.036215	3.39E-02	1.12E-02	14
C6orf106	MI	0.303284	0.073754	3.92E-05	2.82E-01	8
Fgr	MI	-0.09772	0.03101	1.63E-03	3.59E-03	11
RIIAD1	MI	-0.12117	0.031744	1.35E-04	2.18E-02	7
DHX36	MI	-0.24093	0.054141	8.59E-06	2.70E-01	20
RP11-325L7.1	MI	-0.0766	0.018358	3.01E-05	7.49E-01	17
CCDC157	MI	0.068215	0.016904	5.45E-05	3.95E-01	20
GGCX	MI	0.162779	0.028913	1.80E-08	1.79E-01	20
AC009531.2	MI	-0.10116	0.026133	1.08E-04	2.64E-01	9
RNF215	MI	0.064147	0.014112	5.48E-06	9.06E-01	20

It should be noted that the *P*-value from the SMR analysis of one gene did not reach statistical significance after Bonferroni correction. Additionally, two genes failed to pass the HEIDI test.

MI, myocardial infarction. SMR, summary-data-based Mendelian randomization. HEIDI, heterogeneity in dependent instruments. NSNP, number of single nucleotide polymorphism.

**Table 2:** The results of the colocalization analysis between eleven genes and myocardial infarction are presented

Exposure (Gene)	Outcome (Disease)	PP.H0	PP.H1	PP.H2	PP.H3	PP.H4	PP.H3+ PP.H4
MRAS	MI	0.0001	0.0001	0.0001	0.0177	0.9822	0.9999
RP11-378J18.8	MI	0.0001	0.0001	0.0001	0.0251	0.9749	1
C6orf106	MI	0.0001	0.0005	0.0001	0.0764	0.9231	0.9995
Fgr	MI	0.0001	0.0172	0.0001	0.9378	0.0449	0.9827
RIIAD1	MI	0.0001	0.0001	0.0001	0.0253	0.9747	1
DHX36	MI	0.0001	0.0001	0.0001	0.0492	0.9507	0.9999
RP11-325L7.1	MI	0.0001	0.0011	0.0001	0.0249	0.9740	0.9989
CCDC157	MI	0.0001	0.0024	0.0001	0.0776	0.9200	0.9976
GGCX	MI	0.0001	0.0001	0.0001	0.0535	0.9465	1
AC009531.2	MI	0.0001	0.0030	0.0001	0.1231	0.8739	0.9970
RNF215	MI	0.0001	0.0024	0.0001	0.0759	0.9218	0.9976

Notably, colocalization analysis revealed 11 genes all exhibited positive colocalization with MI. This indicates that the eleven genes share genetic variation loci with MI, providing genetic evidence for a robust causal relationship between these genes and the disease. Colocalization was stringently defined as the presence of a shared causal variant supported by posterior probability criteria: either PP4≥0.75 or the sum of PPH3 and PPH4 exceeding 0.8.

MI, myocardial infarction. PP, posterior probability.

### Bulk RNA-Seq analysis of a rat model with ligation of the LAD coronary artery to identify genes with strong causal relationships to MI

**Figure 2:**
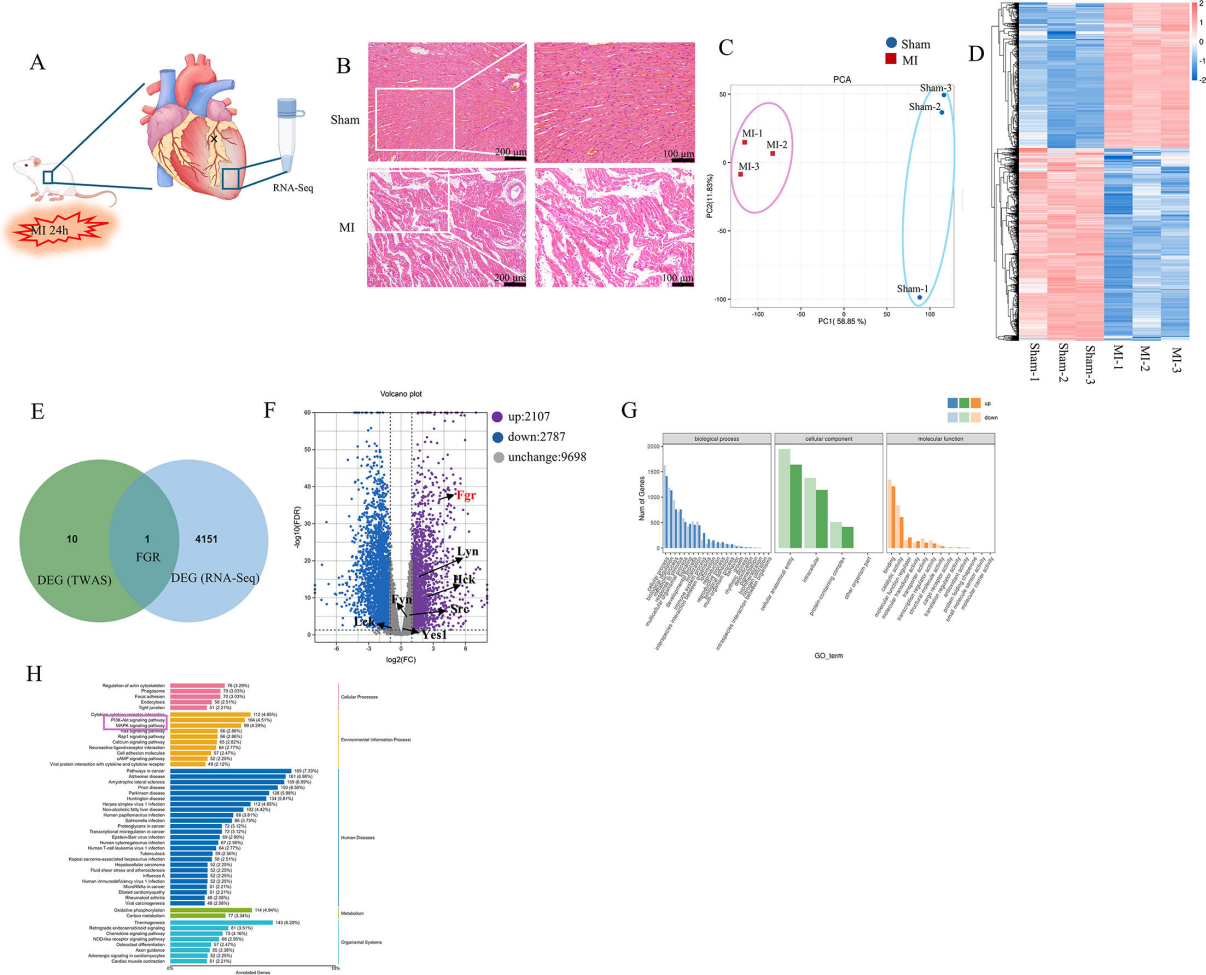
RNA-seq identifies differential gene expression in the apical region of a rat myocardial infarction model. A. The schematic diagram illustrates the establishment of a myocardial infarction model and the collection of RNA sequencing samples. B. HE staining results of rat myocardial tissue demonstrated that cardiomyocytes in the sham-operated group were arranged neatly and compactly with no myofibril breakage, while those in the model group were disorganized and loosely arranged with obvious myofibril breakage, confirming the successful establishment of the myocardial infarction model (*n*=3 rats/group). C. Principal component analysis (PCA) results of sequencing samples (*n*=3 rats/group). D. Cluster analysis results of differentially expressed genes (*n*=3 rats/group). E. By intersecting the differentially expressed genes identified through RNA sequencing analysis with the susceptibility genes identified via TWAS analysis, it was determined that Fgr may serve as a causal gene for MI. F. The differential peaks are illustrated in a volcano plot (|log2 (fold change) |>2 & false discovery rate (fdr)<0.01). G-H Gene Ontology (GO) and Kyoto Encyclopedia of Genes and Genomes (KEGG) enrichment analyses were conducted on the differentially expressed genes to examine the pathways through which the Fgr gene may influence the development of myocardial infarction. The pathways that Fgr may potentially act on are highlighted in the pink frame.

Initially, we verified the successful establishment of the MI model in rats. (Figure 2A–B). Subsequently, we performed bulk RNA-seq analysis on the infarcted apical region, identifying 4894 DEGs, with 2107 genes up-regulated and 2787 genes down-regulated in the model group. To characterize inter-sample heterogeneity, detect outliers, and assess data quality, we performed PCA. The results revealed minimal heterogeneity among samples within groups, indicating that the identified DEGs were robust (Figure 2C). These DEGs were subjected to clustering analysis (Figure 2D). By intersecting these DEGs with the 11 genes identified from the TWAS analysis, we arrived at a single gene of interest—Fgr (Figure 2E–F). Given that Fgr belongs to the SRC family, we examined the expression patterns of other SRC members, including Src, Fyn, Yes, Lck, Lyn, and Hck (Figure 2F). Consistent with the expression trend of Fgr, Lyn, and Hck, they were significantly up-regulated in the model group, while the remaining four genes did not exhibit significant differences between the two groups. Notably, the up-regulation of Fgr was more pronounced compared with the other two genes, and coupled with its identification in the TWAS analysis, we posit that Fgr may play a more pivotal role in the myocardial infarction model than other SRC family members. Furthermore, we conducted Gene Ontology and Kyoto Encyclopedia of Genes and Genomes (KEGG) enrichment analyses on the DEGs to elucidate the potential signaling pathways in which Fgr may be involved, including PI3K-Akt signaling pathway (Figure 2G–H). It is plausible that Fgr participates in the pathophysiological changes of MI through this signaling pathway [30].

### Differential gene expression across cell types elucidated by human cardiac snRNA-seq data

**Figure 3:**
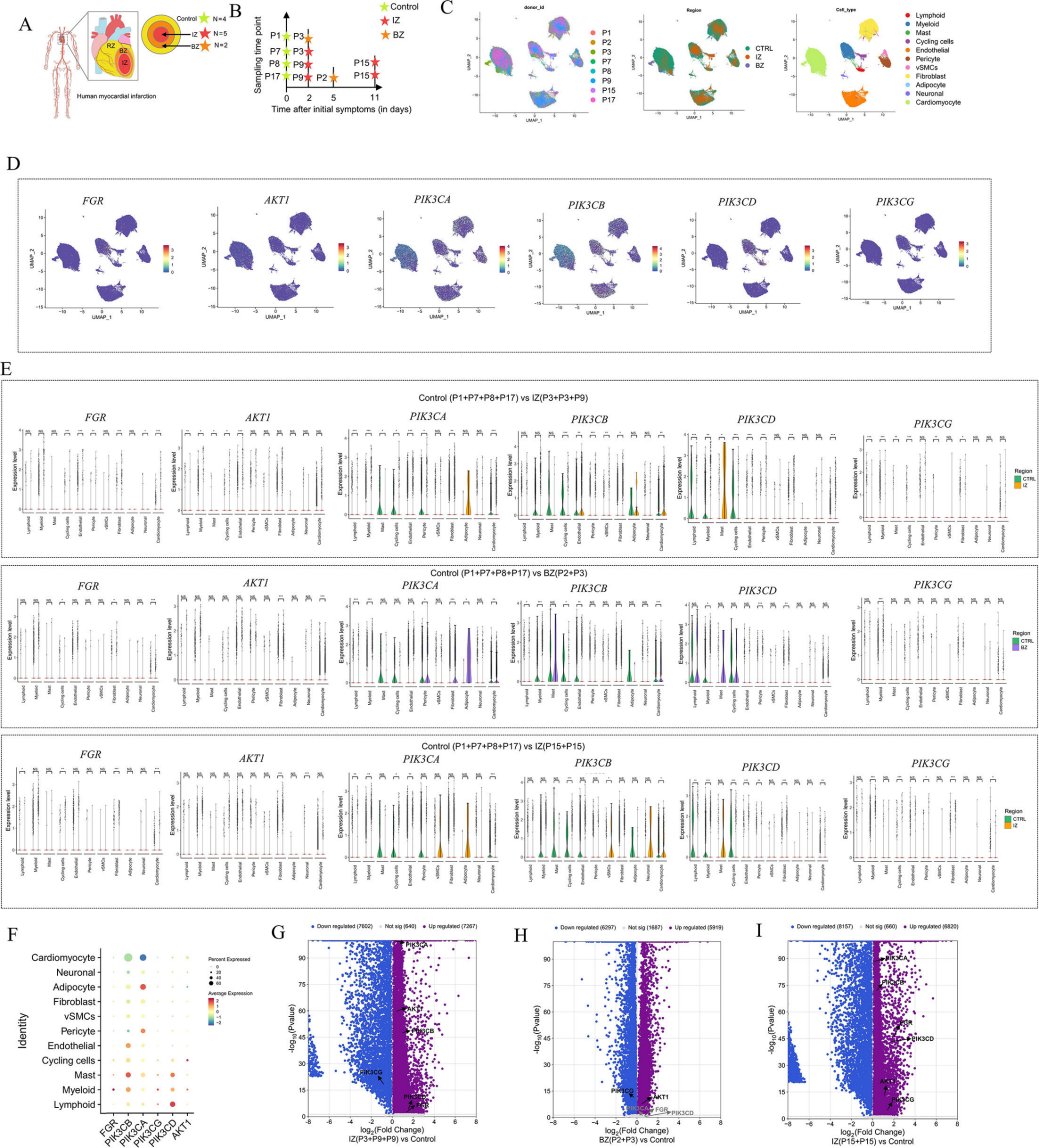
SnRNA-seq data analysis of human MI. A. Study overview. IZ, ischemic zone; BZ, border zone; RZ, remote zone. B. Time points of sampling. P represents the patient number. C. UMAP was used to depict the snRNA-seq data derived from all samples, encompassing a total of 83,582 cells. In this context, VSMCs refer to vascular smooth muscle cells. D. UMAP visualization depicting the distribution of genes in different cell types. E. Violin plots are employed to illustrate the differential expression of the genes Fgr, AKT1, PIK3CA, PIK3CB, PIK3CD, and PIK3CG across different cell types in various groups (CTRL vs. IZ (P3 + P3 + P9), CTRL vs. BZ, and CTRL vs. IZ (P15=p15). F. The bubble plot presents a comprehensive visualization of the proportions of the genes (Fgr, AKT1, PIK3CA, PIK3CB, PIK3CD, and PIK3CG) distributed across various cell types within all samples. G-I. The volcano plot presents the differential gene expression profiles across different groups. Black arrows indicate genes with significant differences in expression between the two compared groups, while light-gray arrows represent genes showing no statistically significant difference in expression. The significance levels are denoted as *P<0.05, **P<0.01, ***P<0.001, and ****P<0.0001, as detailed in the figure legends.

We employed an integrative single-cell genomics approach that combined snRNA-seq and ST, both derived from the same tissue, to map human cardiac cells under homeostatic conditions and following MI. A total of 11 samples were collected from eight individuals, comprising four non-transplanted donor hearts serving as controls, along with samples from tissues containing necrotic regions (IZ and BZ) of patients with AMI (Figure 3A–B). After excluding low-quality nuclei, we successfully obtained 83,582 nuclei from all samples suitable for snRNA-seq. Initially, we grouped the cells based on the integrated snRNA-seq data from all samples, following batch effect correction. The resulting clusters were then labeled using well-curated marker genes sourced from the literature, enabling the identification of ten primary cardiac cell types (Figure 3C). Subsequently, we presented the expression patterns of the target genes across different cell types (Figure 3D). In three groups of MI samples, it was identified that compared with the control group samples, the expression of Fgr was significantly up-regulated in cardiomyocytes, fibroblasts, and cycling cells (Figure 3E–F). Similarly, genes associated with the PI3K/Akt signaling pathway were predominantly up-regulated in inflammatory cells, fibroblasts, and cardiomyocytes. Finally, three volcano plots were used to display the DEGs between different types of samples (Figure 3G–I). The results revealed that the expression levels of both Fgr and PI3K/Akt were significantly up-regulated in the infarcted area.

### Validation of spatial co-localization of Fgr and PI3K/Akt using spatial transcriptomics data

**Figure 4:**
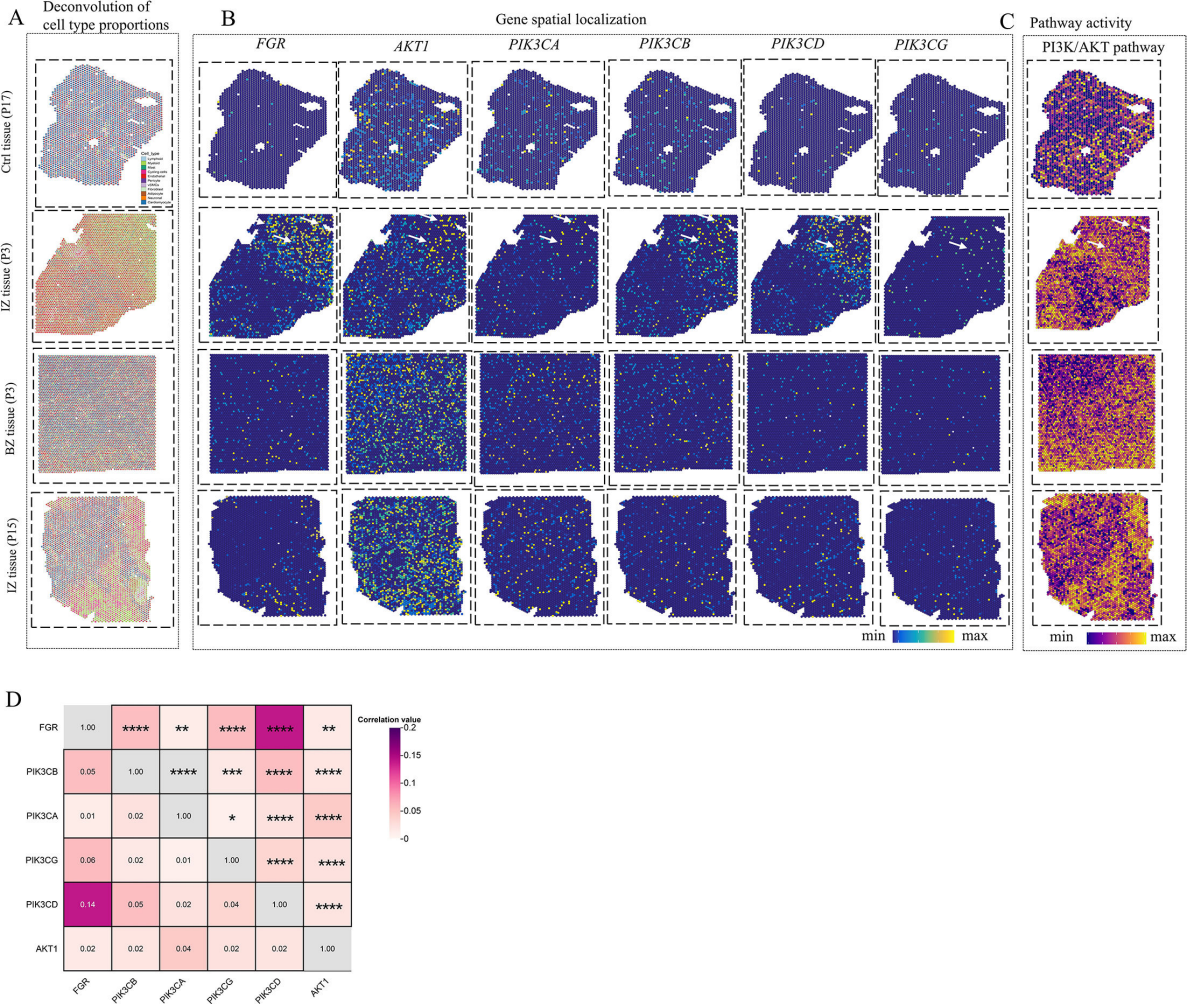
Spatial transcriptomic analysis of human myocardial infarction. A-C. Analyzing spatial transcriptomics data with cell-type deconvolution (**A**), gene spatial localization (**B**) and pathway activity (**C**) for control (CTRL), ischemic zone, and border zone. The spatial co-localization expression patterns of key genes are indicated by white arrows. D. The heatmap displays the correlation coefficients related to the spatial co-localization of key genes. The levels of statistical significance are indicated by the symbols *P<0.05, **P<0.01, ***P<0.001, and ****P<0.0001.

Our study leveraged ST as a powerful tool to elucidate the spatial expression patterns of DEGs. Given that each ST spot encompasses a cluster of cells, we successfully refined the spatial resolution by deconvoluting the cell-type compositions within each spot, utilizing annotated snRNA-seq data from the same biological samples (Figure 4A). The consistency between the estimated cell-type compositions from ST and the observed compositions in snRNA-seq validated the reliability of our deconvolution approach. More importantly, we observed a striking spatial co-localization between genes involved in the PI3K/Akt signaling pathway and Fgr (Figure 4B–D). This compelling finding provided strong support for our hypothesis that Fgr may exert a beneficial effect on cardiac function by enhancing the activity of the PI3K/Akt signaling pathway. Furthermore, we employed the AddModuleScore method to estimate signaling pathway activities for each spot based on spatial gene expression data. By correlating the spatially resolved pathway activities with the estimated cellular abundance at each spot, we established a direct link between spatial cell composition and cellular function at the tissue slide level. For instance, in ischemic regions, we identified a positive correlation between elevated PI3K/Akt signaling activity and an increased abundance of myeloid cells (Figure 4C). These results collectively underscore the potential of integrating ST with pathway analysis to uncover novel mechanisms underlying tissue-specific responses and cellular functions, offering valuable insights for future therapeutic strategies targeting cardiac diseases.

### Unraveling the spatial organization mechanisms of myocardial tissue

**Figure 5:**
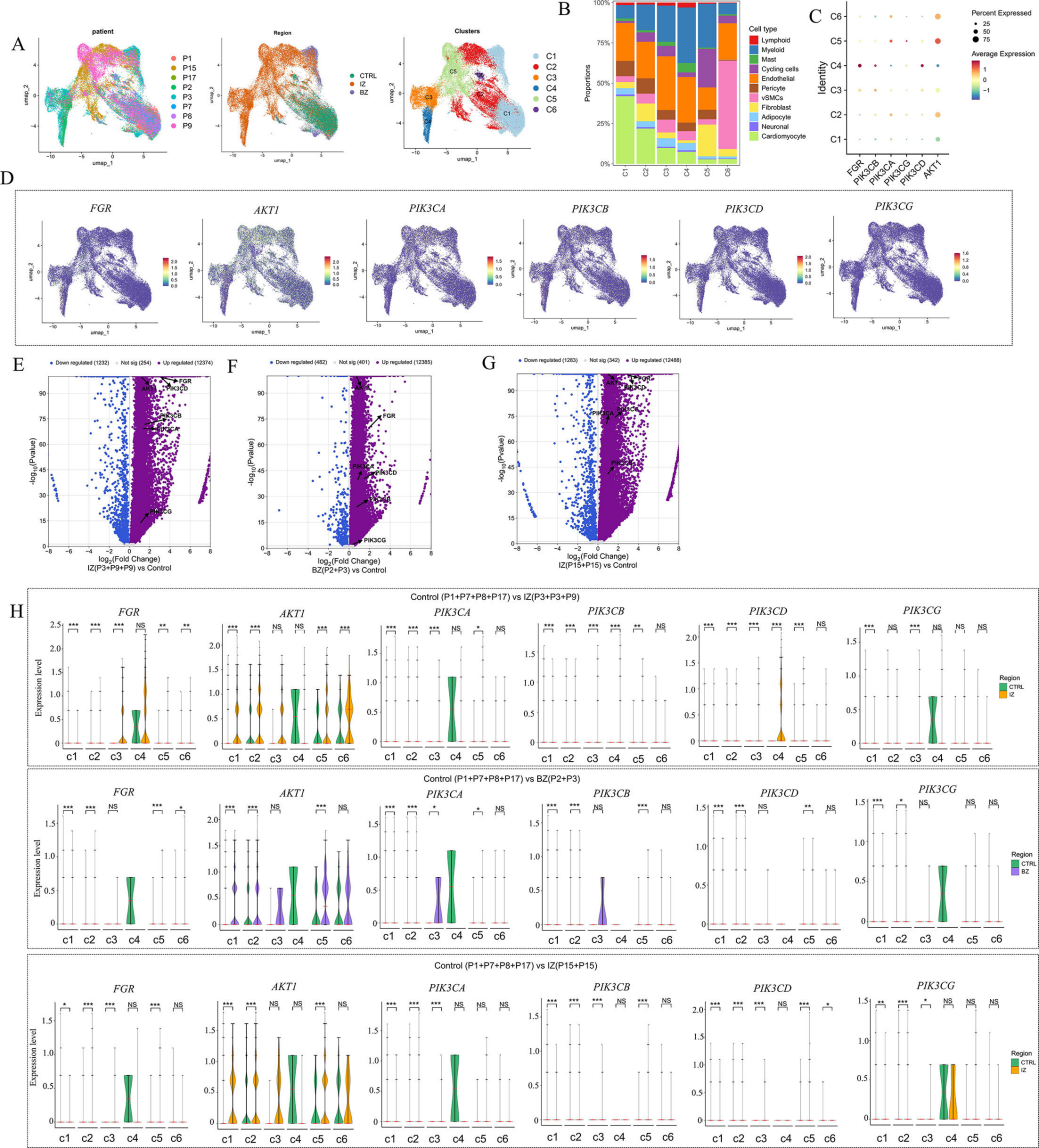
Characterization of tissue organization using spatial transcriptomics data. A.UMAP visualization of spatial transcriptomics spots, constructed based on their cell-type compositions. A-B. The UMAP representation of all patient samples obtained from spatial transcriptomics (**A**), along with an illustration of the relative abundance of major cell types across the myogenic (control and border zone) and ischaemic groups (**B**). C. The bubble plot illustrates the expression profiles of key genes across distinct clusters. D. UMAP visualization of all patient samples concerning the key genes derived from spatial transcriptomics data. E-G. Three volcano plots depict the differentially expressed genes between groups of distinct spatial transcriptomics samples. H. The violin chart depicts the varying expression levels of key genes among various clusters in different groups.

To delve into the spatial organization of myocardial tissue, we harnessed ST data. By conducting unsupervised clustering of spots from all samples based on their cell-type compositions, we successfully identified six clusters, which we subsequently defined as major cell-type niches (Figure 5A). We put forward the hypothesis that these niches act as potential fundamental structural components that are shared across different tissue slides, thereby facilitating comparisons among subjects. Upon visualizing these niches within their spatial context, we discovered interesting associations with the underlying sample conditions. For instance, cell-type niches 1 were uniformly distributed across a control slide, whereas cell-type niches 3, 4, and 5 were localized to distinct regions on an ischemic slide, implying a potential link between the spatial distribution of these niches and the pathological state of the tissue. We then proceeded to test the overrepresentation of annotated cell types derived from snRNA-seq within these cell-type niches. Our analysis revealed the presence of four myogenic cell-type niches (niches 1, 2, 3, and 4), which were enriched with cardiomyocytes, endothelial cells, and pericytes, suggesting their significance in the normal myocardial structure and function (Figure 5B). Additionally, we identified an inflammatory cell-type niche (niche 4) predominantly composed of myeloid and lymphoid cells, indicating an active immune response within the tissue, and a fibrotic cell-type niche (niche 5) containing fibroblasts, myeloid, and lymphoid cells, with the fibrotic niche having a higher proportion of fibroblasts and the inflammatory niche having more myeloid and lymphoid cells. Finally, we observed niches associated with rare cell types in the myocardium, such as cell-type niche 6 linked to VSMCs and cell-type niche 5 also encompassing adipocytes, lymphoid cells, and cycling cells. The integration of our findings offers an exhaustive depiction of cellular co-localization events, thereby empowering subsequent molecular comparisons throughout the entire spectrum of tissue zonations within this atlas (Figure 5C–D). The highly DEGs in each group were visualized using volcano plots (Figure 5E–G). It can be observed that the expression levels of Fgr, AKT1, PIK3CA, PIK3CB, PIK3CD, and PIK3CG were all elevated in the three MI groups. Upon further localizing the DEGs to specific clusters, it was found that Fgr predominantly exhibited differential expression in Clusters 2 and 3 (Figure 5H). Akt1 mainly showed differential expression in Clusters 1, 2, and 5. Additionally, PI3K genes predominantly displayed differential expression in Clusters 1, 2, and 3.

### Exploration of the expression patterns of Fgr and PI3K/Akt in various subtypes of cardiomyocytes

**Figure 6:**
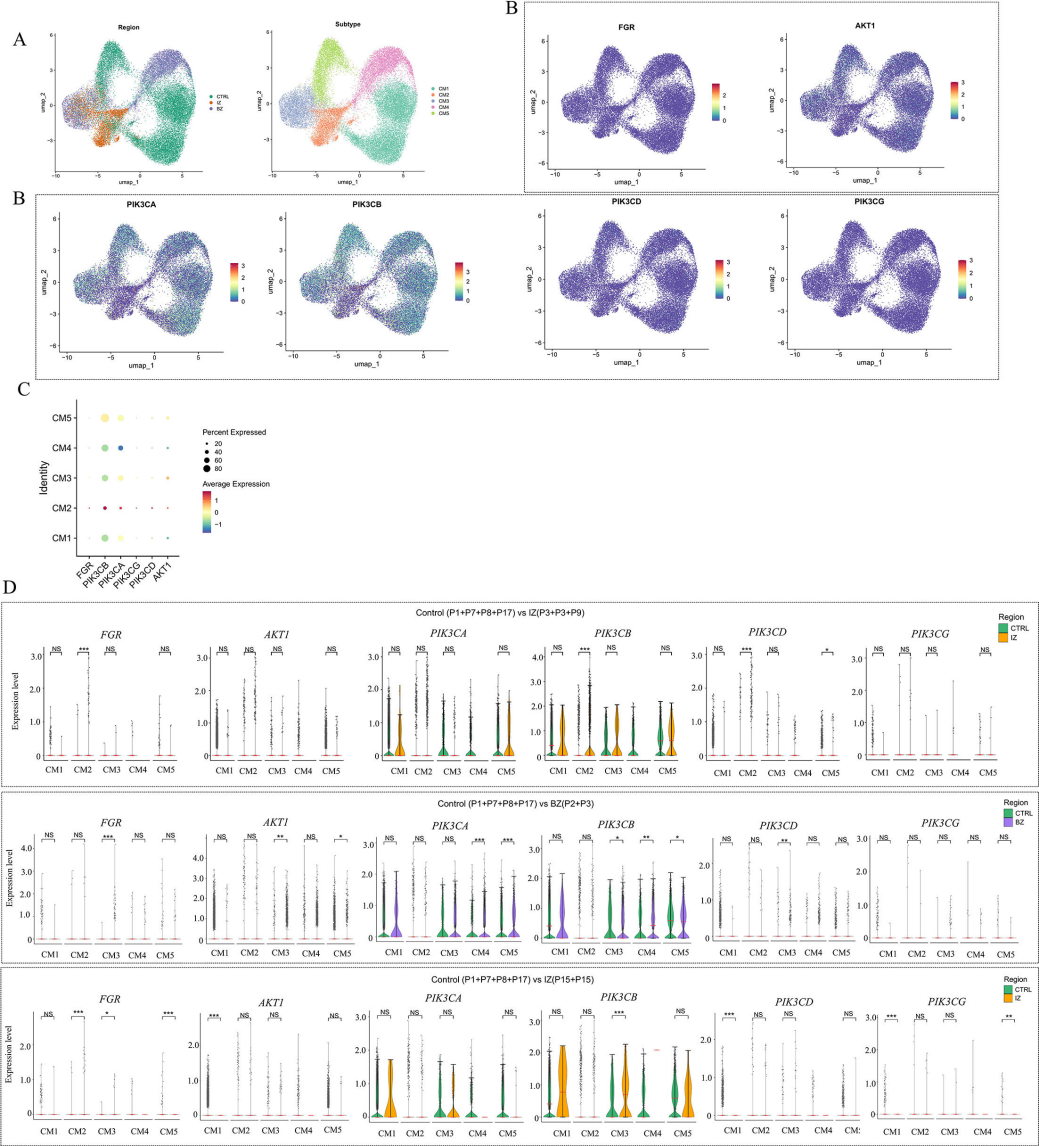
Sub-clustering within cardiomyocytes. A. UMAP of sub-clustering of cardiomyocytes. B. Gene expression of Fgr, AKT1, PIK3CA,PIK3CB,PIK3CD, and PIK3CG. C. A bubble plot illustrates the expression patterns of key genes across different cardiomyocyte sub-populations. D. Violin plots demonstrate whether there are differences in the expression of key genes among different cardiomyocyte sub-populations across groups.

To further delve into the diverse cardiomyocyte states, we endeavored to unravel the molecular heterogeneity of cardiomyocytes following MI. We integrated the snRNA-seq data derived from cardiomyocytes into a unified low-dimensional space and subsequently clustered the cells (Figure 6A). This approach unveiled five distinct cell states of ventricular cardiomyocytes (vCM1-5), which were present across multiple samples and data modalities. These cardiomyocyte states are indicative of different cellular stress conditions during the acute phase of myocardial infarction. Specifically, vCM1 represents a ‘non-stressed’ state, vCM2 signifies a ‘pre-stressed’ state, and vCM3 denotes a ‘stressed’ state [21]. Differential gene expression analysis demonstrated a notable up-regulation of Fgr in vCM2, while PI3K exhibited a distinct up-regulation in both vCM2 and vCM3 (Figure 6B–D). These findings suggest that early intervention to enhance the up-regulation of Fgr and the PI3K/Akt pathway in the vCM2 state could potentially enhance the cardiomyocytes’ ability to adapt to the ischemic environment.

### *In vivo* and *in vitro* validation of Fgr up-regulation in MI model

**Figure 7:**
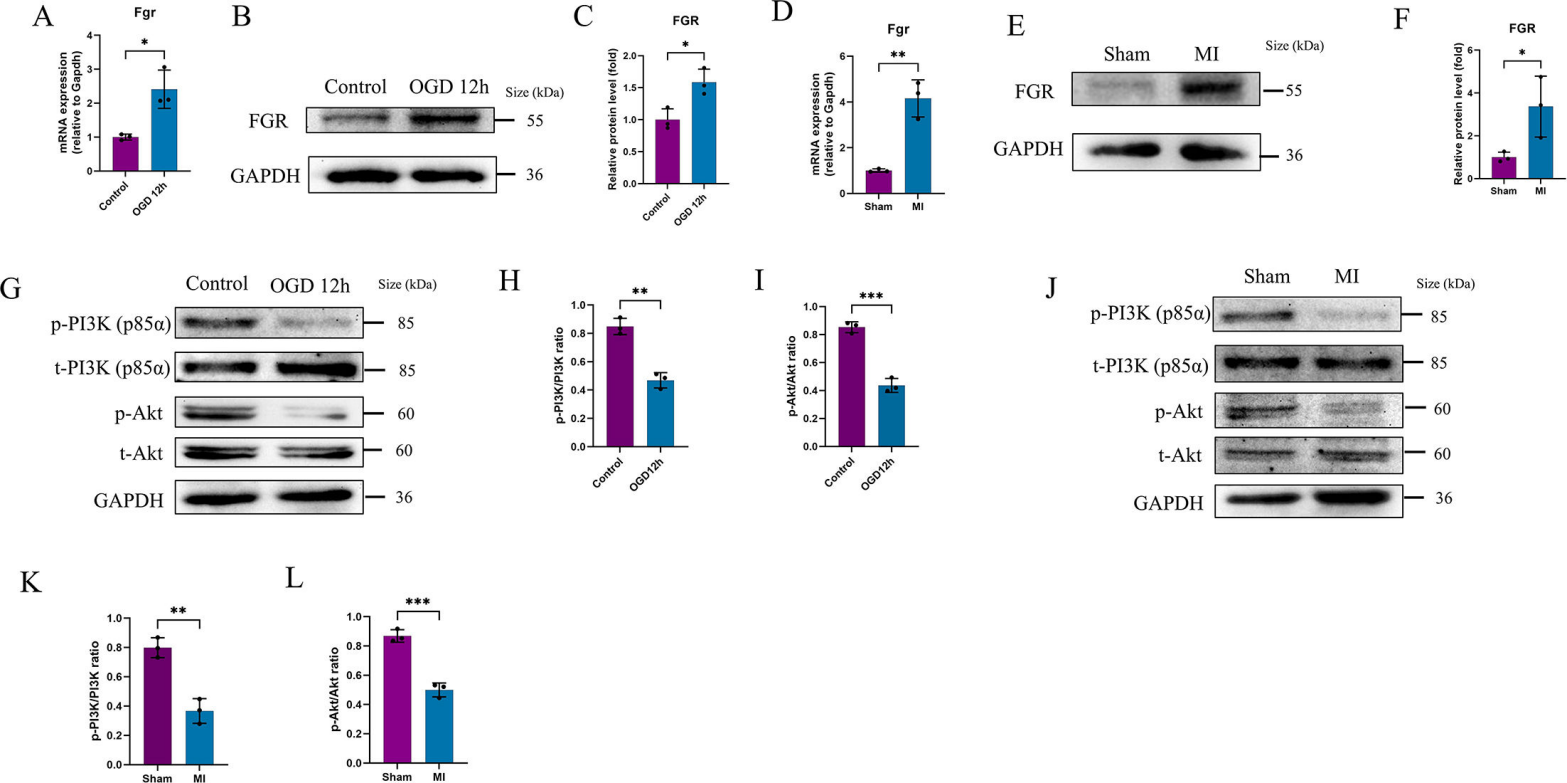
Validation of Fgr gene high expression in MI model: *in vivo* and *in vitro* studies. A. Relative Fgr mRNA expression levels were compared between the NC group and the OGD for 12 h group in H9C2 cells (*n*=3 for each group). B-C. Fgr protein levels in H9C2 cells from the NC group and the OGD for 12 h group were measured using Western blot analysis and quantified with ImageJ software (*n*=3 for each group). D. The relative mRNA expression levels of Fgr were compared between sham and MI group (*n*=3 per group). E-F. Western blot analysis was employed to measure Fgr protein levels between sham and MI group, with subsequent quantification using ImageJ software (*n*=3 per group). G. Western blot analysis was performed to detect the phosphorylation levels of PI3K protein and Akt protein in the NC and OGD model groups (*n*=3 per group). H-I. The subsequent quantification of the phosphorylation levels of PI3K protein and Akt protein. J. Western blot analysis was conducted to assess the phosphorylation levels of PI3K protein and Akt protein in the sham-operated and MI model groups (*n*=3 per group). K-L. The phosphorylation levels of PI3K and Akt proteins were subsequently quantified. *P<0.05, **P<0.01, ***P<0.001, as elaborated in the figure legends. NC, normal control. OGD, oxygen–glucose deprivation.

Through TWAS and RNA-seq analyses, we identified Fgr as a promising candidate gene with significant implications in myocardial infarction. Given that both TWAS and RNA-seq validated Fgr gene expression at the transcriptome level, we sought to further determine whether Fgr protein was up-regulated in the model group. To this end, we employed *in vitro* and *in vivo* models, including H9C2 cardiomyocytes subjected to OGD for 12 h and a rat model of myocardial infarction induced by ligation of the LAD for 24 h. Utilizing PCR and Western blotting techniques, we examined Fgr expression at both the transcriptional and translational levels. Consistent with our previous *in vivo* RNA-seq results, we observed elevated Fgr mRNA levels in the H9C2 OGD model, as well as in the ischemic apical region of the rat heart (Figure 7A and D). Importantly, Fgr protein levels were also significantly increased, indicating that the up-regulation of Fgr is not merely transcriptional but also translates to increased protein abundance (Figure 7B–C and E–F). Similarly, in both cell models and animal models, we validated the reduction in the phosphorylation levels of PI3K and Akt (Figure 7G–L). This provides experimental evidence for our subsequent investigation into whether Fgr protects cardiomyocytes by regulating the phosphorylation levels of the PI3K/Akt signaling pathway.

### Down-regulation of Fgr expression or function promotes myocardial injury by reducing the phosphorylation levels of PI3K/Akt

**Figure 8:**
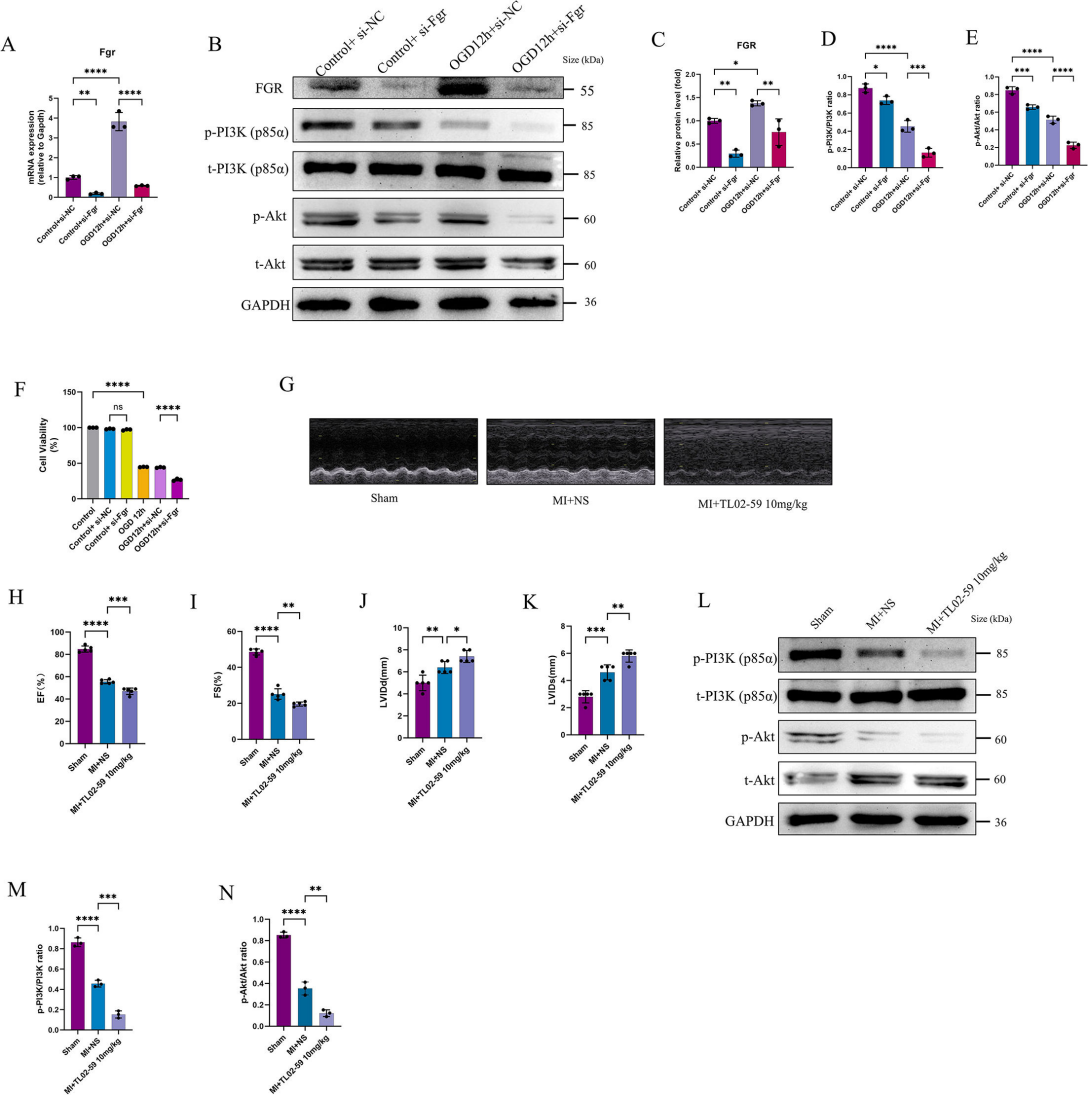
Down-regulation of Fgr expression or function promotes myocardial injury by reducing the phosphorylation levels of PI3K/Akt. A. The knockdown efficiency of siRNA targeting the Fgr gene was evaluated at the mRNA level (*n*=3 for each group). B. The knockdown efficiency of siRNA targeting the Fgr gene was evaluated at the protein level, and we also examined the phosphorylation levels of PI3K and Akt proteins. C-E. The subsequent quantification of the Fgr (**C**) and phosphorylation levels of PI3K protein (**D**) and Akt protein (**E**). F. H9C2 cell viability was measured using the Cell Counting Kit-8 (CCK8) assay. G. Cardiac function of rats in different groups was assessed using echocardiography. Representative M-mode echocardiography images are presented (*n*=5 per group). H-K. Ejection fraction (EF), fractional shortening (FS), left ventricular internal diameter at end-diastole (LVIDd), and left ventricular internal diameter at end-systole (LVIDs). L. Western blot analysis was performed to detect the phosphorylation levels of PI3K/Akt. M-N. The subsequent quantification of the phosphorylation levels of PI3K protein (**M**) and Akt protein (**N**). *P<0.05, **P<0.01, ***P<0.001, ****P<0.0001, as elaborated in the figure legends.

To investigate whether Fgr exerts protective effects on H9C2 cardiomyocytes under OGD, transient Fgr knockdown was achieved via siRNA transfection. First, the efficacy of Fgr silencing was validated at both transcriptional and translational levels (Figure 8A–C). Moreover, we demonstrated that knocking down Fgr led to a reduction in the phosphorylation levels of PI3K/Akt (Figure 8B and D–E). Subsequently, cell viability was assessed using the CCK-8 assay following siRNA treatment. The results revealed that combined OGD (12 h) and Fgr-siRNA intervention significantly attenuated cell survival rates compared with the OGD 12 h + si-NC group (Figure 8F). These findings provide evidence that knocking down Fgr promotes cellular damage by inhibiting the phosphorylation levels of PI3K/Akt. In other words, Fgr exerts a protective effect on cells under OGD conditions, which may be mediated through up-regulating the phosphorylation levels of PI3K/Akt.

To investigate whether Fgr exerts comparable cardioprotective effects *in vivo* to those observed in the OGD model, we utilized a rat LAD coronary artery ligation model. Selective Fgr inhibition was achieved via tail vein injection of TL02-59, followed by echocardiographic evaluation of cardiac function. Quantitative analysis revealed that Fgr suppression significantly compromised key functional parameters, including LVIDd, LVIDs, LVEF, and LVFS (all *P*<0.05 vs. sham) (Figure 8G–K). Crucially, following the inhibition of Fgr function in animals, a significant decrease in the phosphorylation levels of PI3K/Akt was also observed (Figure 8L–N). These data demonstrate that inhibiting Fgr exacerbates cardiac functional impairment in the MI model, which is likely a consequence of the further reduction in PI3K/Akt phosphorylation levels induced by Fgr inhibition.

## Discussion

Timely salvage of jeopardized cardiomyocytes can significantly attenuate cardiac dysfunction, reduce heart failure incidence, and improve long-term prognosis in patients with AMI. Leveraging left ventricular eQTL data from European population cohorts, we employed TWAS analysis coupled with SMR and coloc validation to prioritize 11 susceptibility genes associated with myocardial infarction. To further investigate their differential expression in infarcted myocardium, RNA sequencing of rat apical infarct zones revealed significant up-regulation of Fgr in the ischemic region. Both *in vivo* MI models and *in vitro* OGD assays consistently demonstrated that Fgr up-regulation might alleviate cardiomyocyte injury by regulating the phosphorylation levels of PI3K/Akt, thereby significantly contributing to the preservation of cardiac function post-infarction. These findings not only unravel novel therapeutic targets for AMI intervention but also provide new insights into the pathophysiological processes underlying MI progression.

The PI3K-Akt and MAPK signaling cascades have been extensively studied in MI models. Consistent with our KEGG enrichment analysis of RNA sequencing data, the PI3K-Akt and MAPK signaling pathways were demonstrated to play critical roles in myocardial infarction pathogenesis. Src family kinases (SFKs) exert pleiotropic effects in cellular signal transduction, primarily through interactions with multiple signaling hubs to regulate proliferation, survival, adhesion, migration, and invasion [31]. Notable SFK-modulated pathways include PI3K-Akt, MAPK, and STAT3 signaling axes [32]. Specifically, Src-mediated phosphorylation of PI3K regulatory subunits triggers Akt activation, promoting cell survival and metabolic reprogramming [33]. Constitutively active Src sustains STAT3 phosphorylation at Tyr705, enabling nuclear translocation and transcription of antiapoptotic genes [34]. While SFKs are well-studied, Fgr—a member of this kinase family—has not been directly implicated in MI pathogenesis based on current literature. In tumor-related research, it has been found that Fgr could modulate the phosphorylation levels of PI3K/Akt, offering a mechanistic rationale for future studies aimed at exploring its specific role in the pathogenesis of myocardial infarction [30]. Moreover, we utilized spatial colocalization analysis of ST genes to identify a potential mutual regulation between Fgr and the PI3K/Akt signaling pathway. In addition to Fgr, the SFKs comprise a diverse group of non-receptor tyrosine kinases, including Src, Fyn, Yes, Lck, Lyn, and Hck. These kinases are key mediators of cellular signaling processes and exhibit distinct tissue-specific expression patterns and functional roles. Our RNA sequencing data further revealed up-regulation of Lyn and Hck in infarct zones, alongside Fgr, collectively implicating SFK family members as critical mediators of MI pathophysiology. However, the precise mechanism underlying Frg activation in ischemic myocardium remains elusive and warrants further investigation.

The SFKs emerge as a double-edged sword in atherogenesis. Early investigations demonstrated that hematopoietic deficiency of Hck and Fgr attenuates atherosclerotic plaque formation by abrogating endothelial adhesion and migration. Paradoxically, this deficiency also promotes plaque instability through monocyte subset dysregulation and subendothelial accumulation, raising caution about targeted Src kinase interventions in plaque inflammation [35]. Concurrently, Fgr-deficient macrophages exhibit pronounced antifibrotic phenotypes, suggesting pro-fibrotic activities of Fgr [35]. This raises critical implications for myocardial repair post-infarction, where elevated Fgr expression may exacerbate cardiac remodeling and compromise ventricular function. Collectively, these findings emphasize the intricate functional duality of Src kinases in vascular pathologies, necessitating the development of precision-targeted therapeutic strategies that account for both spatial and temporal expression patterns.

Our study has limitations that warrant acknowledgment. Specifically, we focused exclusively on the early phase of MI and the cardioprotective effects of Fgr during this acute stage. The precise role of Fgr during the critical repair phase post-MI remains undefined, particularly regarding its potential dual functionality in modulating inflammation and fibrosis. Any therapeutic intervention aiming to enhance Fgr expression in the infarct zone must be temporally calibrated to avoid unintended pathological consequences, a hypothesis requiring rigorous validation through staged experimental models. Additionally, all experimental data derive from rodent studies, and the clinical translatability of these findings to human MI patients demands cautious extrapolation. Lastly, it remains uncertain whether Fgr exerts a protective effect on myocardial infarction solely by influencing the phosphorylation levels of the PI3K/Akt signaling pathway. Given the role of Fgr in the field of oncology, we hypothesize that Fgr may also affect the pathophysiological changes of MI through other signaling pathways, such as the STAT or MAPK signaling pathways. This requires further experimental validation. Additionally, there are currently no effective Fgr chemical agonists available to overexpress Fgr or enhance its function, thereby improving myocardial prognosis. However, nanomaterials targeting myocardial tissue to overexpress Fgr may represent an effective strategy, which necessitates extensive validation through animal experiments and human trials. These limitations highlight critical avenues for future investigation to fully elucidate the context-dependent roles of Fgr in cardiovascular pathologies.

In conclusion, this study highlights the crucial role of Fgr in AMI. Fgr is markedly up-regulated during ischemic injury and may protect the heart by modulating PI3K/Akt phosphorylation levels. These novel findings not only establish Fgr as a promising therapeutic candidate but also address a critical knowledge gap in our understanding of ischemic heart disease pathogenesis.

## Supplementary material

online supplementary material 1.

online supplementary material 2.

## Data Availability

The data used in this study is publicly available and can be accessed through the links provided.

## Abbreviations

AMI: acute myocardial infarction
BZ: border zone
CCK-8: Cell Counting Kit-8
CTRL: control
DEGs: differentially expressed genes
GWAS: genome-wide association study
IZ: ischemic zone
KEGG: Kyoto Encyclopedia of Genes and Genomes
LAD: left anterior descending coronary artery
LD: linkage disequilibrium
LVEF: left ventricular ejection fraction
LVFS: left ventricular fractional shortening
LVIDd: left ventricular internal diameter at diastole
LVIDs: left ventricular internal diameter at systole
OGD: oxygen–glucose deprivation
PCA: principal component analysis
PDK1: phosphoinositide-dependent kinase 1
PI3K: phosphoinositide 3-kinase
PIP3: phosphatidylinositol 3,4,5-trisphosphate
RCTD: robust cell-type decomposition
RZ: remote zone
SFKs: Src family kinases
SMR: summary-data-based Mendelian randomization
ST: spatial transcriptomics
TWAS: transcriptome-wide association study
UMAP: uniform manifold approximation and projection
VSMCs: vascular smooth muscle cells
cDNA: complementary DNA
eQTL: expression quantitative trait loci
qPCR: quantitative PCR
