# Supplementary material for: High expression of Fgr in the left ventricle attenuates myocardial injury in the infarcted region via regulating the phosphorylation level of PI3K/Akt
Source: Biosci Rep. 2025 Oct 13;45(10):615–33. doi: 10.1042/BSR20253737 (PMC12784349; doi:10.1042/BSR20253737)
Supplement: online supplementary material 1. [file bsr-45-10-BSR20253737-s001.pdf]

Figure 2

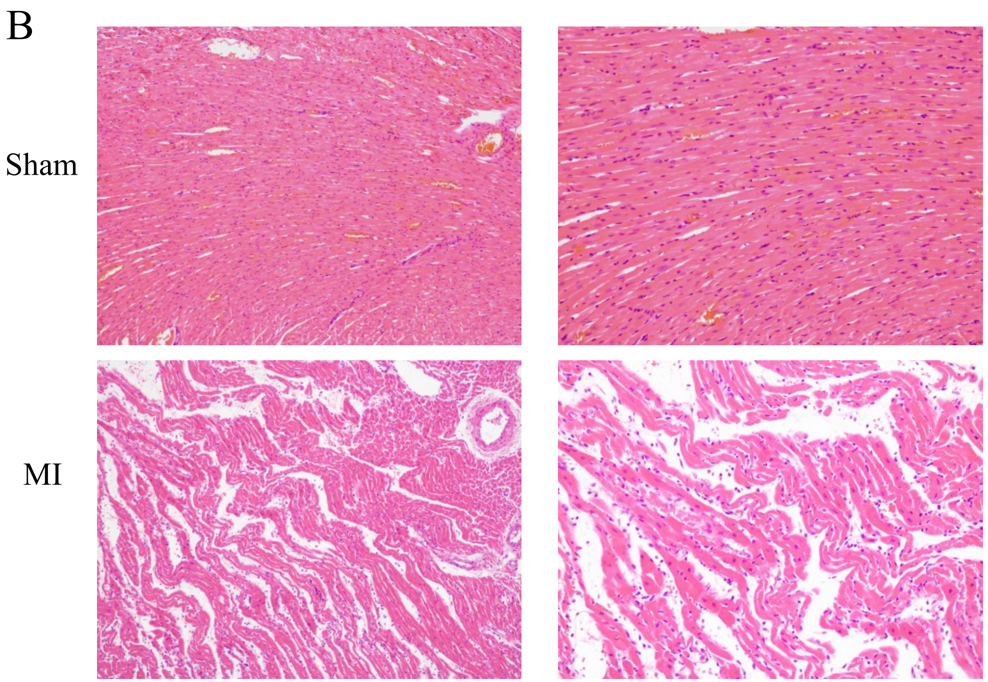

HE-stained images of rat myocardial tissue sections, where the left images are at 100× and the right at 200× magnification.

Figure 3

A

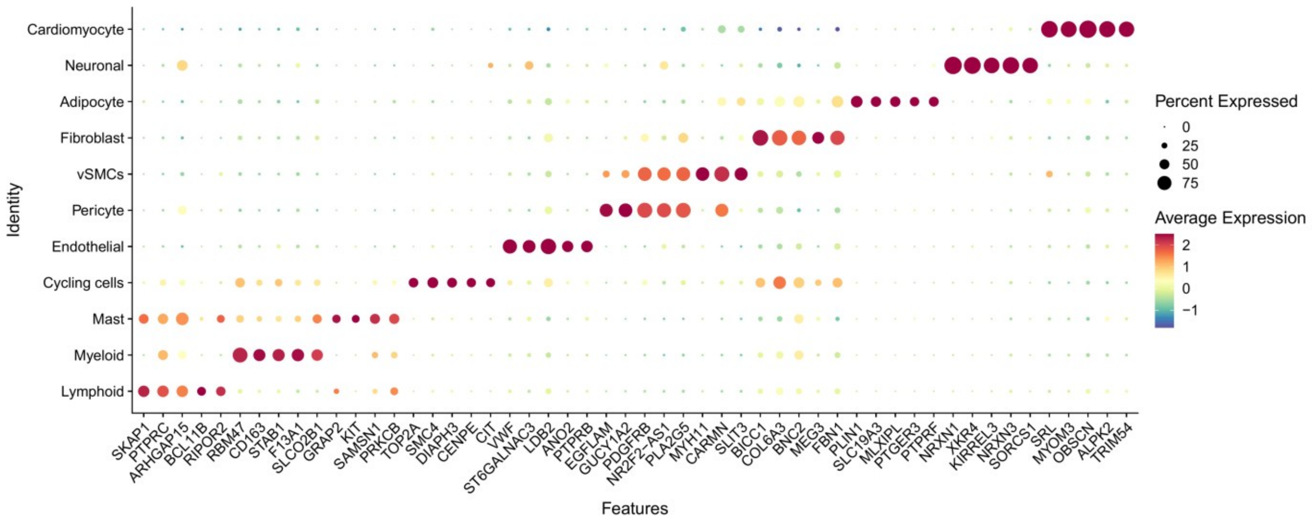

Eleven major cell types were defined using classic marker genes, including lymphocytes, myeloid cells, mast cells, proliferating cells, endothelial cells, pericytes, vascular smooth muscle cells, fibroblasts, adipocytes, neuronal cells, and cardiomyocytes. The single-cell dataset with these well-defined cell types will serve as a reference dataset to assist in identifying cell distribution within spatial transcriptomics data.

Figure 5

A

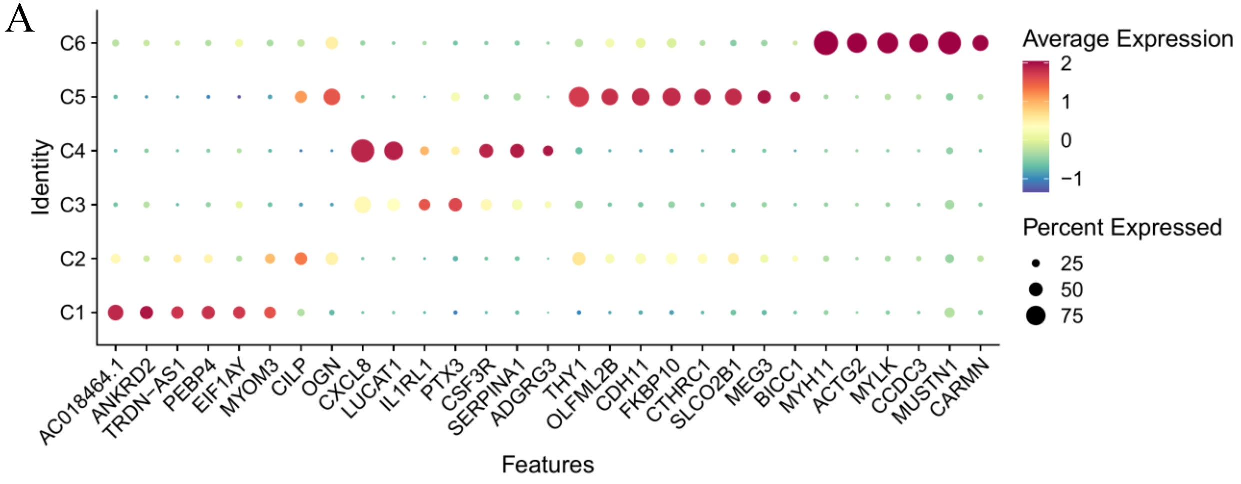

The FindAllMarkers function is utilized to analyze highly expressed genes within each cluster, and genes that are significantly and differentially highly expressed in each cluster are filtered based on the threshold criteria of avg_log2FC > 0.5 & p_val_adj < 0.15.

Figure 6

A

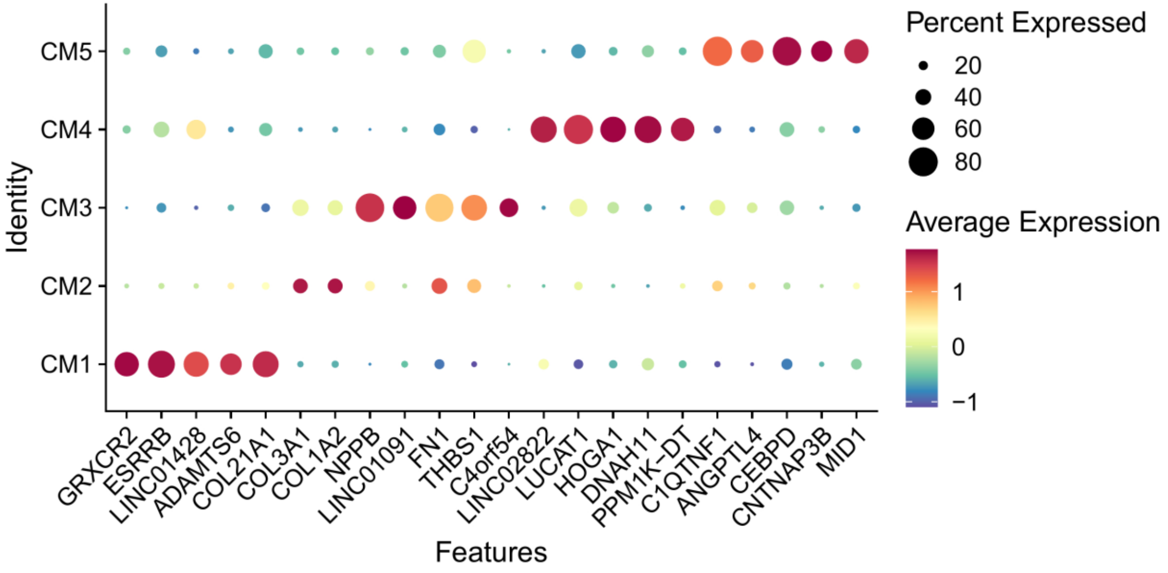

The FindAllMarkers function (with parameters only.pos = TRUE, min.pct = 0.2, and min.diff.pct = 0.2) was employed to identify differentially expressed genes among various cardiomyocyte subpopulations. The top 5 genes with the highest diff.pct (calculated as pct.1 - pct.2) were selected as marker genes for each subpopulation, and a bubble plot was generated to visualize the results.

Figure 7

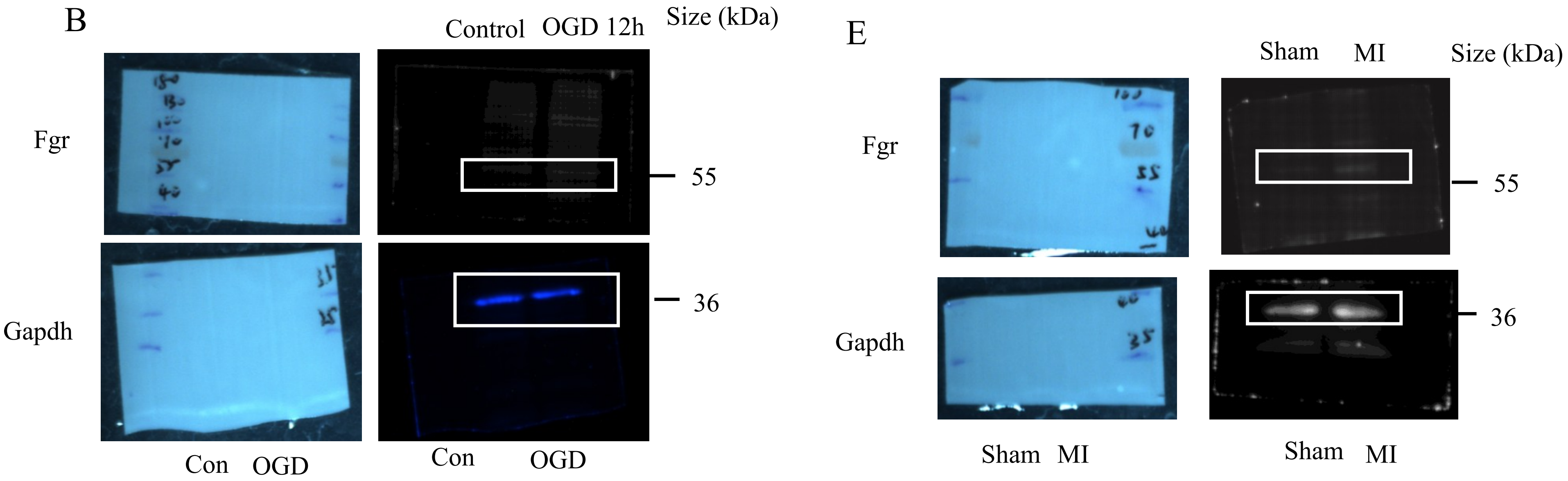
B
Control OGD 12h
Size (kDa)
Fgr
55
Gapdh
36
Con OGD
Con OGD
E
Sham MI
Size (kDa)
Fgr
55
Gapdh
36
Sham MI
Sham MI

Figure 7

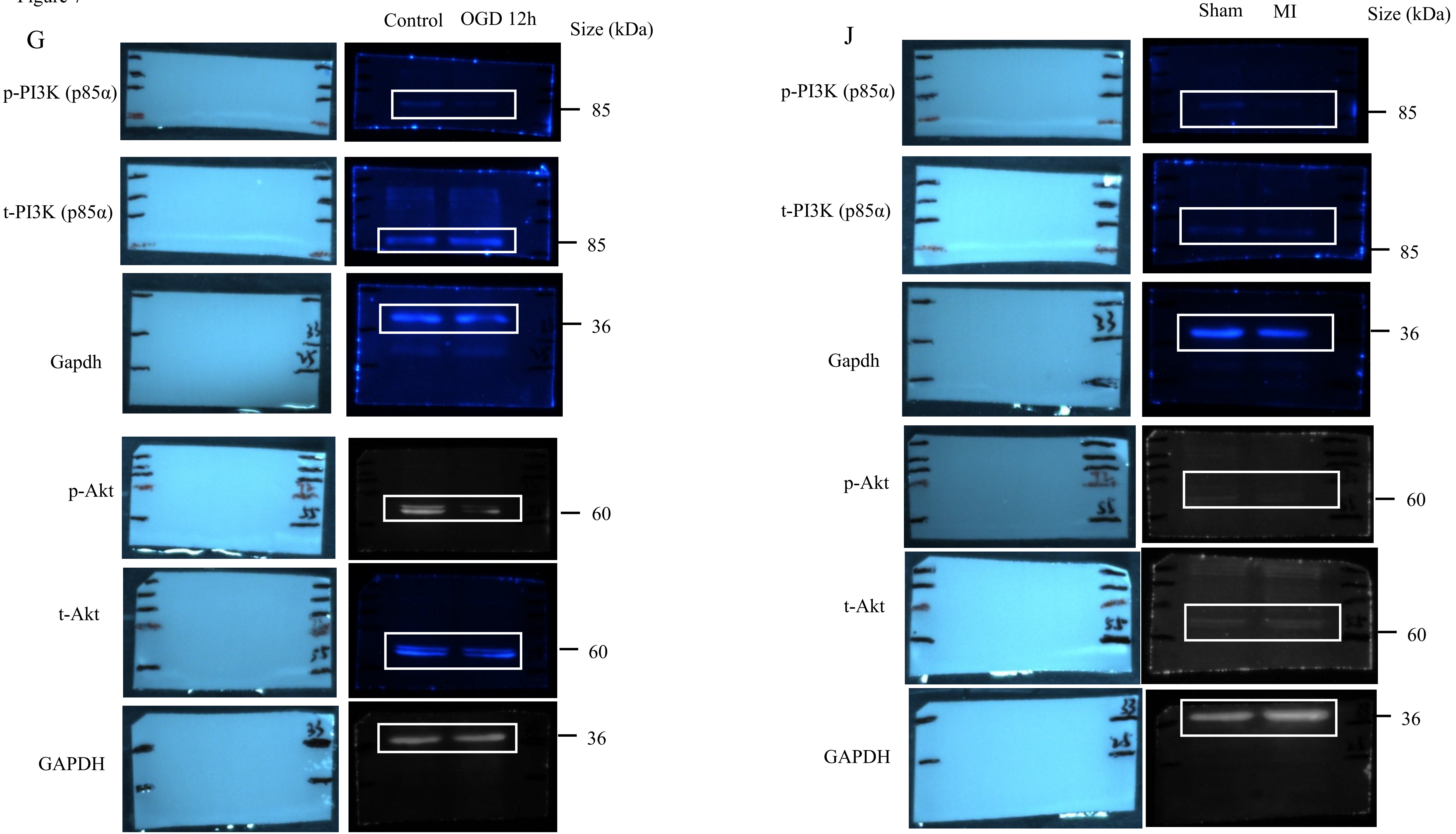
G
Control
OGD 12h
Size (kDa)
p-PI3K (p85α)
85
t-PI3K (p85α)
85
Gapdh
36
p-Akt
60
t-Akt
60
GAPDH
36
J
Sham
MI
Size (kDa)
p-PI3K (p85α)
85
t-PI3K (p85α)
85
Gapdh
36
p-Akt
60
t-Akt
60
GAPDH
36

Figure 8

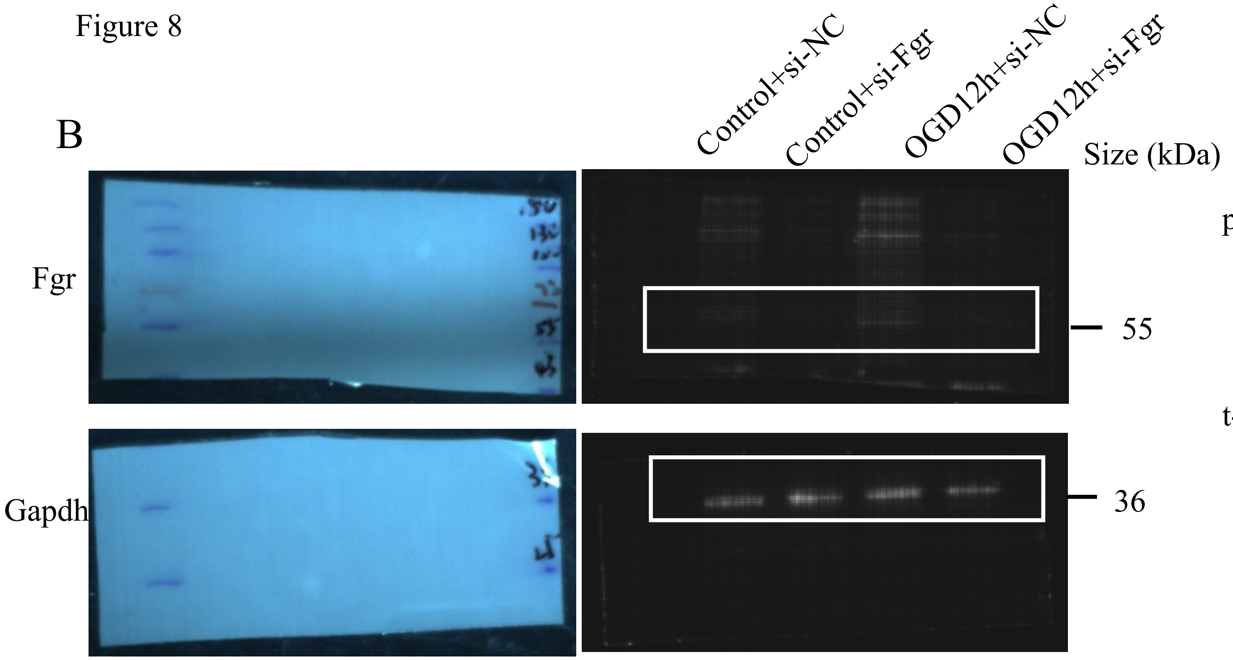
B
Control+si-NC
Control+si-Fgr
OGD12h+si-NC
OGD12h+si-Fgr
Size (kDa)
Fgr
55
Gapdh
36

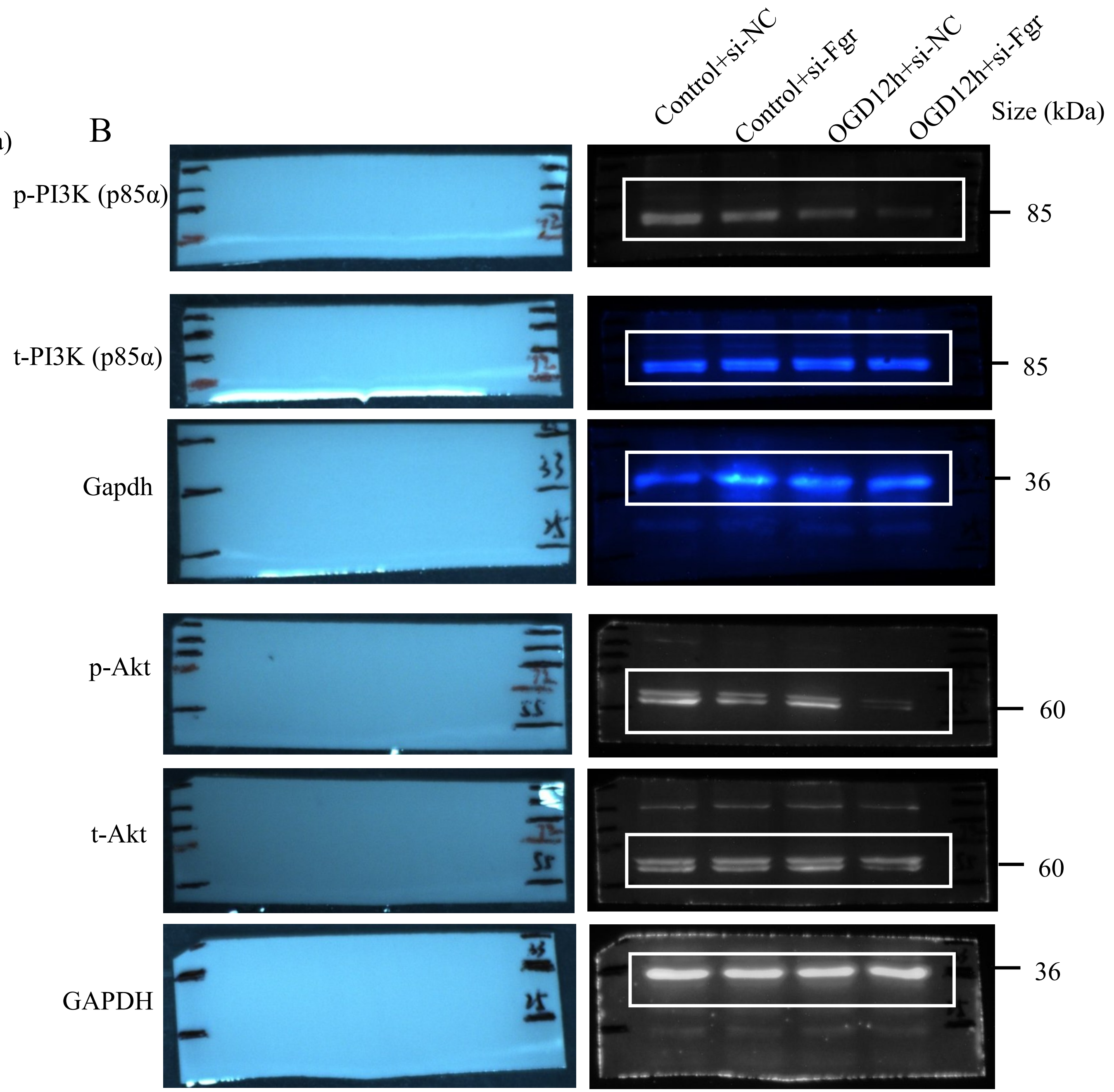
B
Control+si-NC
Control+si-Fgr
OGD12h+si-NC
OGD12h+si-Fgr
Size (kDa)
p-PI3K (p85α)
85
t-PI3K (p85α)
85
Gapdh
36
p-Akt
60
t-Akt
60
GAPDH
36

Figure 8

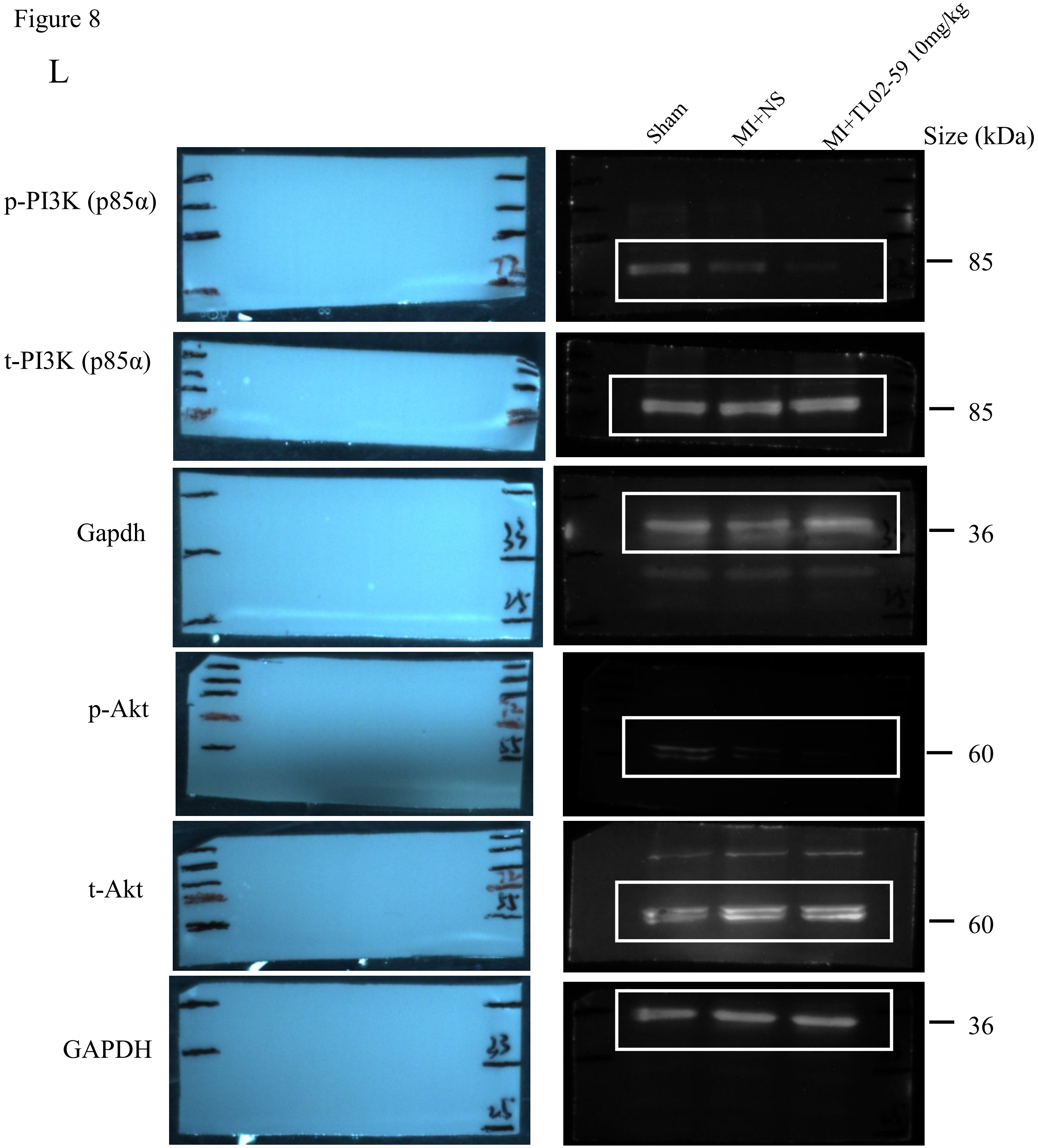
L
Sham
MI+NS
MI+TL02-59 10mg/kg
Size (kDa)
p-PI3K (p85α)
t-PI3K (p85α)
Gapdh
p-Akt
t-Akt
GAPDH
85
85
36
60
60
36
